# Senp7 deficiency impairs lipid droplets maturation in white adipose tissues *via* Plin4 deSUMOylation

**DOI:** 10.1016/j.jbc.2024.107319

**Published:** 2024-04-25

**Authors:** Jingwen Pei, Dayuan Zou, Lu Li, Lulu Kang, Minli Sun, Xu Li, Qianyue Chen, Danning Chen, Bin Qu, Xiang Gao, Zhaoyu Lin

**Affiliations:** 1 State Key Laboratory of Pharmaceutical Biotechnology, Ministry of Education Key Laboratory of Model Animal for Disease Study, Jiangsu Key Laboratory of Molecular Medicine, Model Animal Research Center, National Resource Center for Mutant Mice of China, Nanjing Drum Tower Hospital, School of Medicine, Nanjing University, Nanjing, China; 2 Anhui Province Key Laboratory of Basic and Translational Research of Inflammation-Related Diseases, First Affiliated Hospital of Bengbu Medical University, Bengbu, Anhui, China; 3 Institutes for Systems Genetics, Frontiers Science Center for Disease-Related Molecular Network, National Clinical Research Center for Geriatrics, West China Hospital, Sichuan University, Chengdu, China; 4 Biophysics, Center for Integrative Physiology and Molecular Medicine (CIPMM), School of Medicine, Saarland University, Homburg, Germany

**Keywords:** deSUMOylation, Senp7, lipid storage, lipid droplet, Plin4

## Abstract

Lipid metabolism is important for the maintenance of physiological homeostasis. Several members of the small ubiquitin-like modifier (SUMO)-specific protease (SENP) family have been reported as the regulators of lipid homeostasis. However, the function of Senp7 in lipid metabolism remains unclear. In this study, we generated both conventional and adipocyte-specific *Senp7* KO mice to characterize the role of Senp7 in lipid metabolism homeostasis. Both *Senp7*-deficient mice displayed reduced white adipose tissue mass and decreased size of adipocytes. By analyzing the lipid droplet morphology, we demonstrated that the lipid droplet size was significantly smaller in *Senp7*-deficient adipocytes. Mechanistically, Senp7 could deSUMOylate the perilipin family protein Plin4 to promote the lipid droplet localization of Plin4. Our results reveal an important role of Senp7 in the maturation of lipid droplets *via* Plin4 deSUMOylation.

Lipid metabolism is tightly regulated to maintain energy homeostasis. Several lipogenesis-associated proteins are regulated by a family of sentrin/SUMO-specific proteases (SENPs) (1, 2, 3, 4), suggesting an emerging regulatory mechanism in lipid metabolism. The SENP family regulates the balance between the small ubiquitin-like modifier (SUMO) conjugation and deconjugation (5). The SUMO modification is a reversible and highly dynamic posttranslational modification system that regulates cellular processes by controlling the activity, localization, or stability of proteins (6, 7). A recent study showed that Senp1 can upregulate peroxisome proliferator activated receptor gamma, which is a key regulator of lipid metabolism and adipogenesis, by deSUMOylizing Sharp-1 (2). Senp2 has also been found to deSUMOylate CCAAT enhancer-binding proteins, another key regulator of adipogenesis, to prevent it from ubiquitin-dependent degradation (1). Senp7 has been linked to tumorigenesis, DNA repair, and cytosolic DNA sensing (8, 9, 10, 11). However, the role of Senp7 in lipid metabolism remains largely unknown.

Lipid droplet (LD) is a dynamic lipid storage organelle that serves as storage depots and sources of essential substrates for multiple cellular processes (12). Both inadequate and excessive storage of neutral lipids in LDs are associated with human diseases, such as lipodystrophy, nonalcoholic fatty liver disease, atherosclerosis, and obesity (13, 14, 15). LDs are composed of a core of neutral lipids surrounded by a phospholipid monolayer and associated proteins (16, 17). The regulation of LD-associated proteins is important for the biogenesis, maturation, and degradation of LDs (18, 19, 20).

The perilipin (Plin) family proteins, as the well-characterized LD-coated proteins, can mediate the fusion of LDs and the access of the cytoplasmic lipolytic proteins to LDs (21, 22, 23). Specifically, Plin1 promotes the growth of LDs by markedly increasing Fsp27-mediated directional neutral lipid transfer from smaller to larger LDs (21), whereas Plin2, Plin3, and Plin4 constitute a ready reservoir of coat proteins to permit rapid packaging of newly synthesized triglyceride (TG) and to maximize energy storage during nutrient excess (24, 25, 26, 27). Plin5 controls lipolysis in oxidative tissues, including skeletal muscle, heart, and brown adipose tissue (BAT) (28, 29). Mutations of Plins are associated with metabolic diseases such as lipodystrophy or coronary artery disease (30, 31, 32).

In this study, we determined the role of Senp7 in lipid metabolism by generating *Senp7* conventional and adipocytes specific KO mice. Results showed that *Senp7* deficiency led to decreased fat accumulation in white adipose tissues (WATs) due to impaired LDs maturation in adipocytes. Mechanistically, Senp7 could deSUMOylate Plin4 to promote the maturation of LDs.

## Results

### *Senp7* KO mice show less fat accumulation in WATs

To investigate the physiological role of Senp7, we generated *Senp7* conventional KO mice (*Senp7* KO mice, Fig. S1*A*). The disruption of *Senp7* in mice was confirmed by quantitative real-time polymerase chain reaction (qRT-PCR) and immunoblotting (Fig. S1, *B* and *C*).

**Figure 1:**
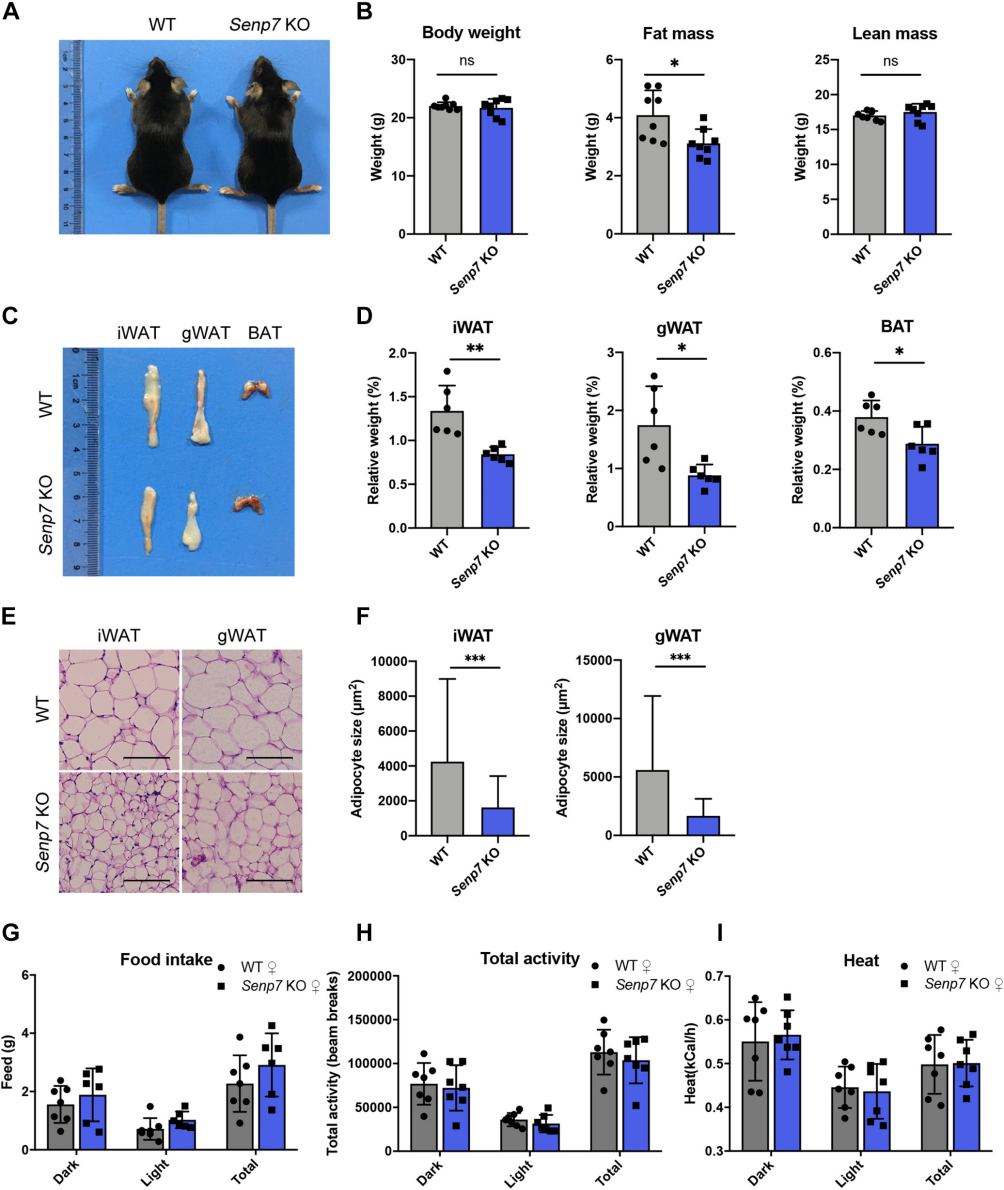
***Senp7* KO mice show less fat accumulation in white adipose tissues.***A*, gross morphology of 14-week-old female mice. *B*, body composition of female WT and *Senp7* KO mice measured by DEXA after 14 weeks on a regular chow diet. n = 8; mean ± SD; ∗*p* < 0.05; ns, no significance by two-tailed *t* test. *C* and *D*, dissection image (*C*) and weight normalized to body weight of organs (iWAT, gWAT, BAT) (*D*) of 14-week-old female WT and *Senp7* KO mice. n = 6; mean ± SD; ∗*p* < 0.05, ∗∗*p* < 0.01; ns, no significance by two-tailed *t* test. *E*, H&E staining of iWAT and gWAT sections from 14-week-old female WT and *Senp7* KO mice. The scale bars represent 100 μm. *F*, statistics of adipocyte size from iWAT and gWAT of 14-week-old female WT and *Senp7* KO mice. Three slices from each tissue with three mice per genotype were analyzed. n = 4500 cells; mean ± SD; ∗∗∗*p* < 0.001 by Mann–Whitney test. *G*–*I*, indirect calorimetry of 14-week-old male WT and *Senp7* KO mice. *G*, feed: food intake. *H*, total activity. *I*, heat: heat generation. n = 7; mean ± SD; ns, no significance by two-tailed *t* test. The *column chart* represents an average value during the light cycle (8:00∼20:00) and dark cycle (20:00∼8:00). BAT, brown adipose tissue; DEXA, dual-energy X-ray absorptiometry; gWAT, gonadal white adipose tissue; iWAT, inguinal white adipose tissue.

There were no significant differences in morphology and body weight between 14-week-old female *Senp7* KO mice and WT littermates when fed a chow diet (Fig. 1, *A* and *B*). Dual energy X-ray absorptiometry (DEXA) scan revealed that compared to littermate control female *Senp7* KO mice exhibited reduced fat mass at 14 weeks of age (Fig. 1*B*). Male *Senp7* KO mice showed similar phenotypes (Fig. S1*D*). Further examination of tissues revealed reduced inguinal and gonadal fat pads in *Senp7* KO mice (Fig. 1, *C* and *D*). The average size of adipocytes in *Senp7* KO mice was significantly reduced in both inguinal white adipose tissue (iWAT) and gonadal white adipose tissue (gWAT, Fig. 1, *E* and *F*), although the adipose tissues still predominantly comprised unilocular adipocytes. The total DNA content of adipose tissues showed no significant difference between WT and *Senp7* KO mice, indicating a similar cell number in the adipose tissues of KO mice (Fig. S1*E*). These data suggest that the decrease in fat mass was primarily caused by a reduction in the size of adipocytes.

*Senp7* KO mice displayed a reduction in interscapular BATs (Fig. 1*D*), while visible LDs were nearly absent in BAT of KO mice (Fig. S1*F*). However, the liver weights did not differ between the genotypes of chow-fed animals (Fig. S1*G*). No ectopic lipid depositions were observed in the H&E-stained liver sections from *Senp7* KO mice (Fig. S1*H*). Moreover, there was no difference in serum TG levels between the two groups (Fig. S1, *I* and *J*).

To investigate the metabolic consequences of global ablation of *Senp7*, 14-week-old KO mice were phenotyped in metabolic cages. The results showed no significant differences in food intake, activity levels, or energy expenditure levels between *Senp7* KO mice and their littermate controls (Figs. 1, *G*–*I* and S2). Taken together, conventional deletion of *Senp7* leads to a reduction in fat mass and a decrease in adipocyte size without causing notable systemic metabolic dysfunction.

### Adipose-specific *Senp7* KO mice have reduced WAT mass

The above data suggests that the elimination of *Senp7* in all tissues of mice results in a reduction of adipose tissue while maintaining a consistent global energy balance. Thus, we hypothesized that the diminished fat mass was caused by the dysfunction of *Senp7* in WATs. To examine the hypothesis, we crossed *Senp7*-floxed (*Senp7*^fl/fl^) mice with *Adipoq*-cre mice to generate adipose-specific KO mice (*Senp7* adipose-specific knockout (AKO) mice). The specificity of *Senp7* deletion in adipose tissues was confirmed by qRT-PCR and immunoblotting (Fig. S3, *A* and *B*).

**Figure 2:**
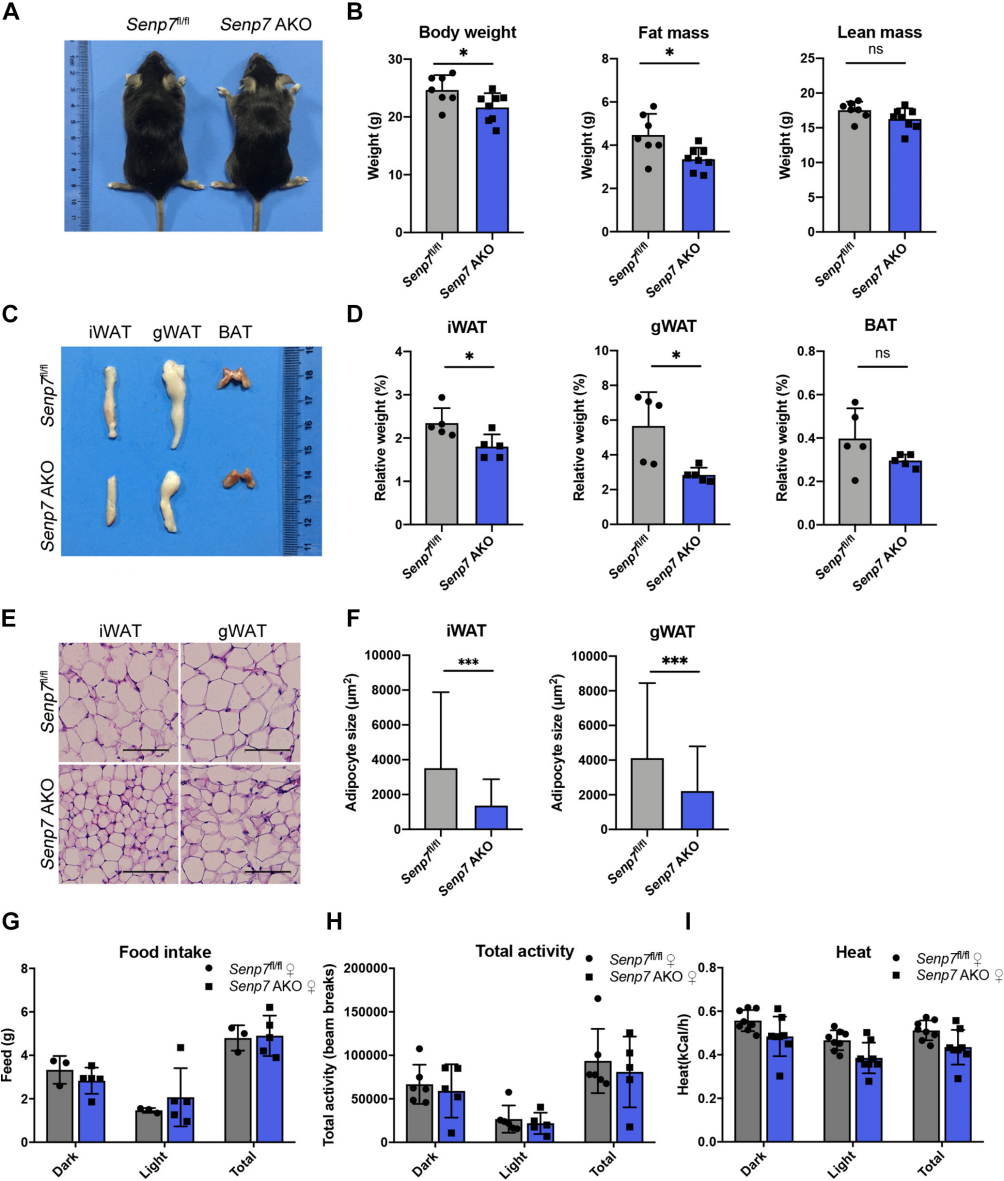
**Adipose-specific *Senp7* KO mice have reduced white adipose tissue mass.***A*, gross morphology of 14-week-old female mice. *B*, body composition of female *Senp7*^fl/fl^ and *Senp7* AKO mice measured by DEXA after 14 weeks on a regular chow diet. *Senp7*^fl/fl^ n = 7, *Senp7* AKO n = 8; mean ± SD; ∗*p* < 0.05; ns, no significance by two-tailed *t* test. *C* and *D*, dissection image of organs (iWAT, gWAT, BAT) of 14-week-old female *Senp7*^fl/fl^ and *Senp7* AKO mice (*C*) and weight normalized to body weight (*D*). n = 5; mean ± SD; and ∗*p* < 0.05, ∗∗*p* < 0.01 by two-tailed *t* test. *E*, H&E staining of iWAT and gWAT sections from 14-week-old female *Senp7*^fl/fl^ and *Senp7* AKO mice. The scale bars represent 100 μm. *F*, statistics of adipocyte size from iWAT and gWAT of 14-week-old female *Senp7*^fl/fl^ and *Senp7* AKO mice. Three slices from each tissue with three mice per genotype were analyzed. n = 4500 cells; mean ± SD; and ∗∗∗*p* < 0.001 by Mann–Whitney test. *G*–*I*, indirect calorimetry of 14-week-old male *Senp7*^fl/fl^ and *Senp7* AKO mice. *G*, feed: food intake. *H*, total activity. *I*, heat: heat generation. n = 8; mean ± SD; and ns, no significance by two-tailed *t* test. The *column chart* represents an average value during the light cycle (8:00∼20:00) and dark cycle (20:00∼8:00). BAT, brown adipose tissue; DEXA, dual energy X-ray absorptiometry; iWAT, inguinal white adipose tissue; gWAT, gonadal white adipose tissue; SENP, sentrin/SUMO-specific protease.

Compared to *Senp7*^fl/fl^ littermates, *Senp7* AKO mice exhibited no obviously abnormal morphology (Fig. 2*A*). At 14 weeks of age, *Senp7* AKO mice showed a significant decrease in fat mass, while no difference in lean mass was found (Figs. 2*B* and S3*C*). Consistently, smaller iWAT and gWAT were observed in *Senp7* AKO mice (Fig. 2, *C* and *D*), indicating that the absence of *Senp7* in adipose tissues impaired fat storage in mice. The histological analysis of iWAT and gWAT in *Senp7* AKO mice revealed a significant reduction in adipocyte size (Fig. 2, *E* and *F*). The total DNA content of iWAT and gWAT suggested a comparable number of adipocytes between *Senp7* AKO and *Senp7*^fl/fl^ mice (Fig. S3*D*).

There was no significant difference of BAT weights between the two groups (Fig. 2, *C* and *D*). Moreover, the histological appearance revealed similar lipid accumulation in BAT of *Senp7*^fl/fl^ mice and AKO mice (Fig. S3*E*).

**Figure 3:**
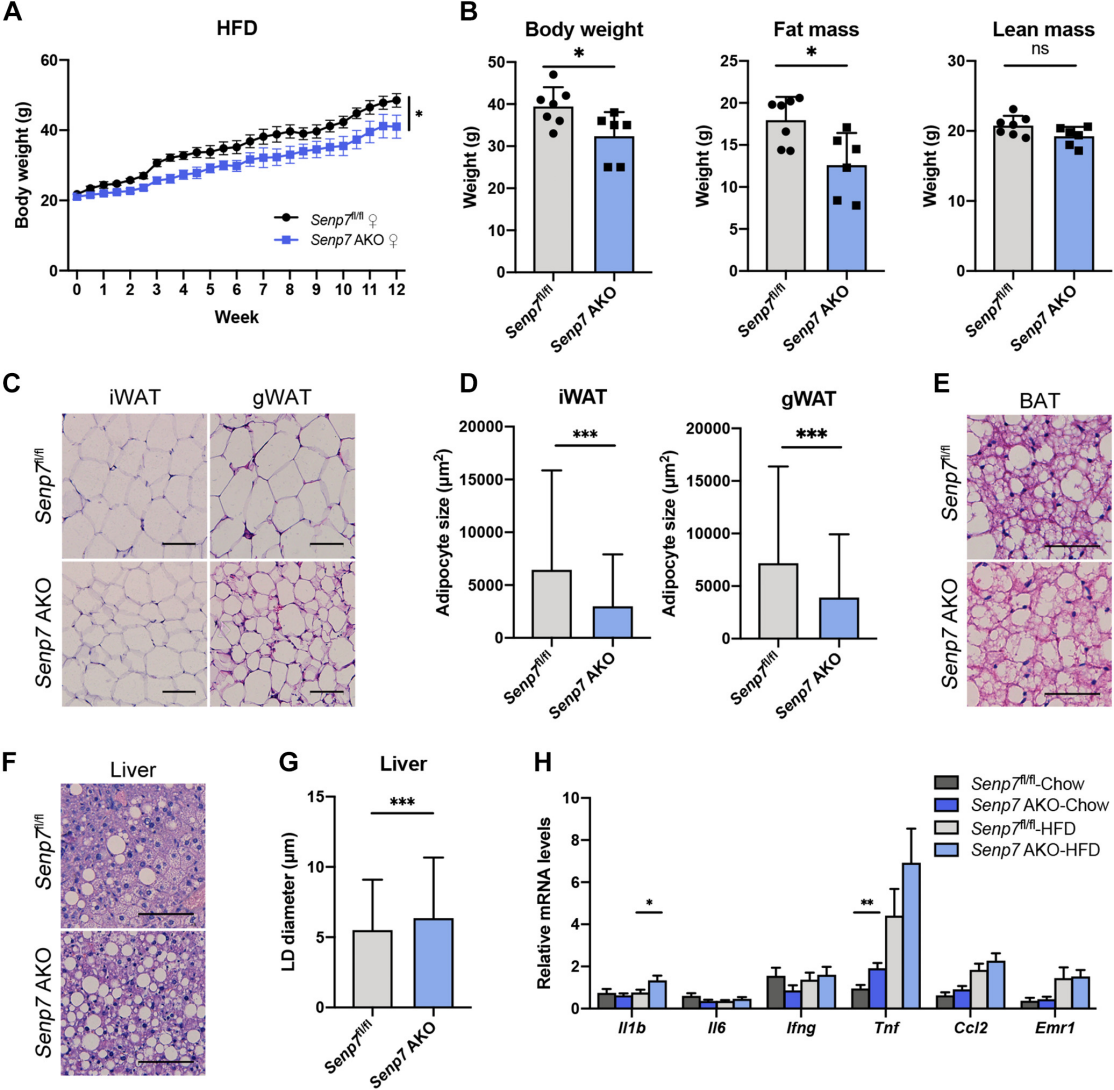
**Adipose-specific *Senp7* KO mice resist to HFD-induced weight gain.***A*, growth curve of female *Senp7*^fl/fl^ and *Senp7* AKO mice during 12-week HFD feeding. n = 6. Error bars are represented as mean ± SEM. Area under curve was analyzed using two-tailed *t* test. ∗*p* < 0.05. *B*, body composition of female *Senp7*^fl/fl^ and *Senp7* AKO mice measured by DEXA after 8-week HFD feeding. n = 6; mean ± SD; ∗*p* < 0.05; and ns, no significance by two-tailed *t* test. *C*, H&E staining of iWAT and gWAT sections from female *Senp7*^fl/fl^ and *Senp7* AKO mice after 12-week HFD feeding. The scale bars represent 100 μm. *D*, statistics of adipocyte size from iWAT and gWAT of mice described in *C*. Three slices from each tissue with three mice per genotype were analyzed. n = 1800 cells; mean ± SD; and ∗∗∗*p* < 0.001 by Mann–Whitney test. *E*, H&E staining of BAT sections from female *Senp7*^fl/fl^ and *Senp7* AKO mice after 12-week HFD feeding. The scale bars represent 50 μm. *F*, H&E staining of liver sections from female *Senp7*^fl/fl^ and *Senp7* AKO mice after 12-week HFD feeding and statistics of the size of lipid droplets in liver. The scale bars represent 100 μm. *G*, statistics of adipocyte size from the LD size in livers described in *F* were measured. Three slices from each mouse with three mice per genotype were analyzed. Mean ± SD;∗∗∗*p* < 0.001 by Mann–Whitney test. *H*, the expression of *Il1b*, *Il6*, *Ifng*, *Tnf*, *Ccl2*, and *Emr1* in WAT from *Senp7*^fl/fl^ and *Senp7* AKO mice was analyzed by real-time quantitative PCR. Expression levels of target genes were normalized to *Rplp0* (alias *36b4*), and data were normalized to *Senp7*^fl/fl^-chow value. Comparisons were performed between *Senp7*^fl/fl^ and *Senp7* AKO mice fed with each diet. n = 6 for *Senp7*^fl/fl^-chow and *Senp7* AKO-chow; n = 8 for *Senp7*^fl/fl^-HFD and *Senp7* AKO-HFD; mean ± SEM; and ∗*p* < 0.05, ∗∗*p* < 0.01 by two-tailed *t* test. BAT, brown adipose tissue; DEXA, dual energy X-ray absorptiometry; gWAT, gonadal white adipose tissue; HFD, high-fat diet; iWAT, inguinal white adipose tissue; SENP, sentrin/SUMO-specific protease.

To confirm the role of Senp7 in lipid accumulation, we challenged the mice with a high-fat diet (HFD) for 12 weeks. Following the attenuated body weight gain (Fig. 3*A*), DEXA measurement at 8 weeks after HFD feeding revealed a significant decrease in fat mass of *Senp7* AKO mice compared to *Senp7*^fl/fl^ mice (Fig. 3*B*), while there were no differences in the absolute lean mass between the two groups (Fig. 3*B*). Histological analysis of iWAT and gWAT indicated that HFD-fed *Senp7* AKO mice exhibited smaller adipocytes than *Senp7*^fl/fl^ mice (Fig. 3, *C* and *D*). This suggests an impairment in the ability of Senp7-deficient adipocytes to enlarge in response to fat overload. However, the morphology of BAT exhibited similarity between groups (Fig. 3*E*). These findings suggest that the specific knockout of Senp7 in adipose tissues impairs the capacity for lipid accumulation in white adipocytes.

We subsequently investigated the effects of *Senp7* AKO on energy expenditure. There were no significant differences in food intake, activity levels, or oxygen consumption between the genotypes (Figs. 2, *G*–*I* and S3, *J*–*L*). Taken together, these findings indicate that the depletion of *Senp7* in adipose tissues results in decreased WAT without inducing systemic metabolic changes, suggesting that Senp7 may contribute to the development of adipose tissues.

### Adipose-specific *Senp7* KO mice showed increased lipid accumulation in the liver

Lipodystrophy is often accompanied by lipid depositions, possibly resulting from enhanced hepatic lipid accumulation driven by the lack of appropriate adipose depots (33). Thus, we analyzed the mice that were fed either chow diets or HFD. The livers of *Senp7* AKO mice fed with chow diets did not exhibit distinct phenotypic variations compared to *Senp7*^fl/fl^ mice (Fig. S3, *F* and *G*). Furthermore, no significant difference in serum TG levels was observed in *Senp7* AKO mice fed with chow diets (Fig. S3, *H* and *I*). However, *Senp7* AKO mice that were fed a HFD for 12 weeks exhibited a disorganized morphology of livers characterized by a significant increase in both the number and size of LDs in comparison to *Senp7*^fl/fl^ mice (Fig. 3, *F* and *G*). HFD challenge did not lead to differences in metabolic indexes in serum of *Senp7*^fl/fl^ and *Senp7* AKO mice (Fig. S4, *A*–*G*). Compared to *Senp7*^fl/fl^ mice, *Senp7* AKO mice exhibited significantly upregulated *Il1b* expression, following 12 weeks of HFD feeding. There was a non-statistically significant increase in the expresion of *Tnf* (Fig. 3*H*). This indicated the increased chronic inflammation in WAT of *Senp7* AKO mice. Overall, our data suggest that adipose-specific loss of *Senp7* may partially prevent the mice from the HFD-induced fat storage but at the expense of ectopic lipid accumulation in the liver to maintain systemic lipid homeostasis.

### *Senp7* deficiency results in reduced size of LDs

**Figure 4:**
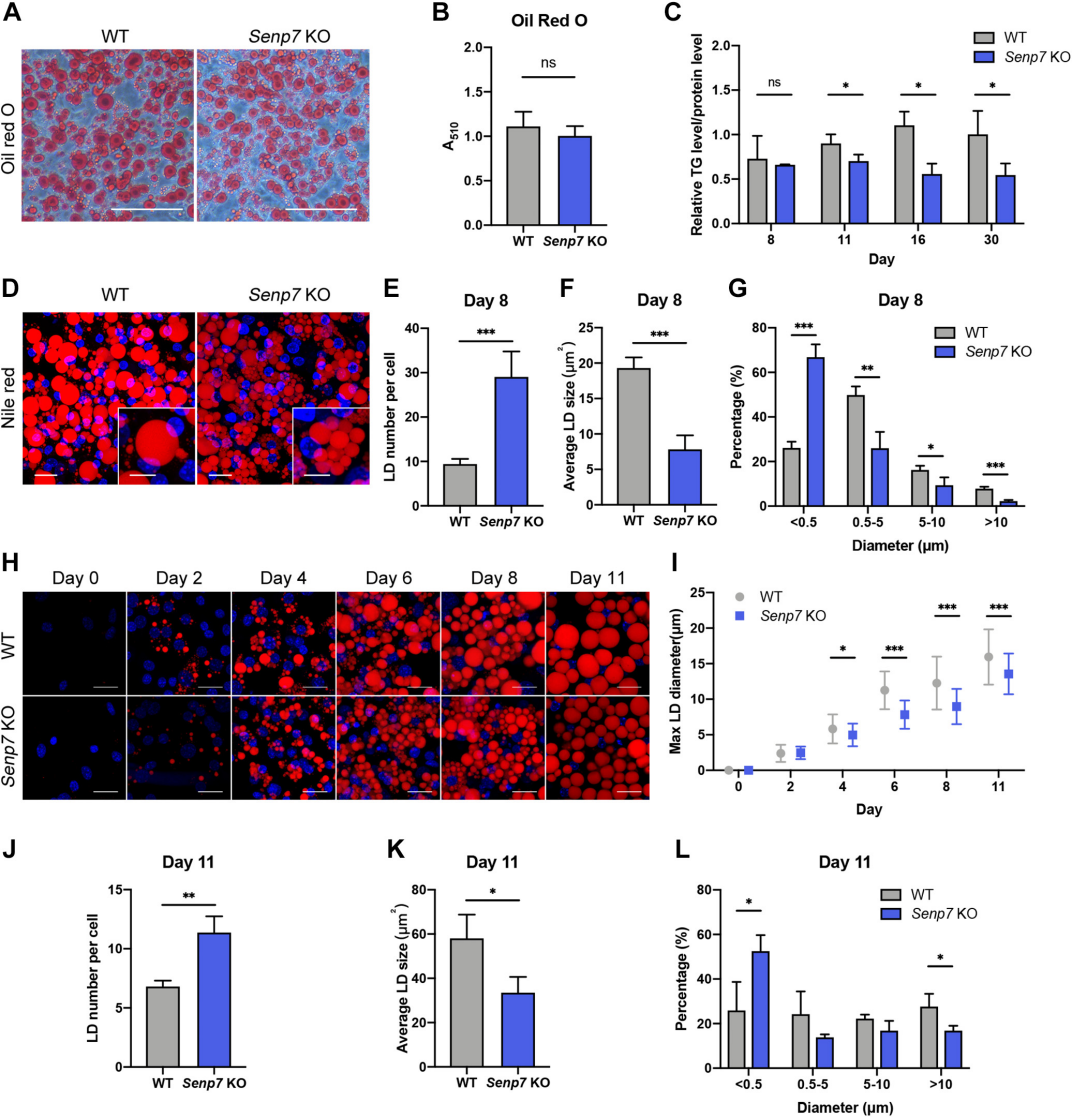
***Senp7* deficiency results in reduced size of LDs.***A*, Oil Red O staining of differentiated primary preadipocytes on day 8. The scale bars represent 100 μm. *B*, the Oil Red O staining in (*A*) was quantitatively measured by A_510_ absorbance analysis. n = 3; mean ± SD; and ns, no significance by two-tailed *t* test. *C*, total TG levels of differentiated primary preadipocytes on indicated day were measured and were normalized to total protein levels. n = 3 on day 8. n = 4 on day 11, 16, and 30. Mean ± SD; ∗*p* < 0.05; and ns, no significance by two-tailed *t* test. *D*–*G*, Nile red staining of differentiated primary preadipocytes on day 8 and quantification of diameter and number of the LDs in 50 cells for each independent experiment (n = 3). The scale bars for main images represent 20 μm; the scale bars for zoomed-in images represent 10 μm. Mean ± SD; ∗*p* < 0.05, ∗∗*p* < 0.01, ∗∗∗*p* < 0.001; and ns, no significance by two-tailed *t* test. *H* and *I*, Nile red of differentiated primary preadipocytes on day 0, 2, 4, 6, 8, and 11 and quantification of diameter of the largest LDs in each cell. The scale bars represent 20 μm. n = 50; mean ± SD; ∗*p* < 0.05, ∗∗∗*p* < 0.001; and ns, no significance by two-tailed *t* test. *J*–*L*, quantification of diameter the LDs in 40 differentiated primary preadipocytes for each independent experiment (n = 3) on day 11. Mean ± SD; ∗*p* < 0.05, ∗∗*p* < 0.01, ∗∗∗*p* < 0.001; and ns, no significance by two-tailed *t* test. All experiments were performed at least twice. LD, lipid droplet; SENP, sentrin/SUMO-specific protease; TG, triglyceride.

To characterize the role of Senp7 in the regulation of adipogenesis, primary preadipocytes were differentiated *in vitro*. After 8 days of adipogenic differentiation treatment, the differentiated KO adipocytes showed more dispersed LDs than WT controls (Fig. 4*A*). Although there was no significant difference in the total level of Oil Red O staining or the concentration of TG in the differentiated adipocytes between the groups at day 8 (Fig. 4, *B* and *C*), prolonged differentiation revealed that *Senp7* KO cells exhibited significantly impaired TG concentration after day 11 (Fig. 4*C*), suggesting reduced lipid accumulation in *Senp7* KO cells.

The aberrant development of LDs may impair the storage of lipid in white adipocytes. To gain further insight into the morphological phenotypes of LDs in *Senp7* KO adipocytes, we systematically assessed the number and size of LDs by utilizing Nile red staining to visualize the neutral lipids (Fig. 4*D*). Indeed, *Senp7* KO adipocytes contained significantly smaller but a higher number of LDs than the control adipocytes (Figs. 4, *E* and *F*). Compared to the control cells, the percentage of mature LDs (>10 μm) was significantly decreased in *Senp7* KO cells, and the percentage of immature LDs (<0.5 μm) (18) in KO cells was significantly increased (Fig. 4*G*). In addition, *Senp7* KO adipocytes showed delayed enlargement of LDs throughout differentiation (Figs. 4, *H* and *I* and S4, *H*–*J*). By maintaining the differentiated adipocytes until day 11 to facilitate the continued LDs growth, we found that the prolonged lipid accumulation partially restored the LDs size distribution pattern (Fig. 4*L*). Still, it could not recover the impaired TG levels and LD volume on day11, 16, and 30 (Figs. 4*C* and S4, *H*–*J*). Overall, our data suggest that *Senp7* deficiency reduces the size of LDs in adipocytes.

### Plin4 is deSUMOylated by Senp7

**Figure 5:**
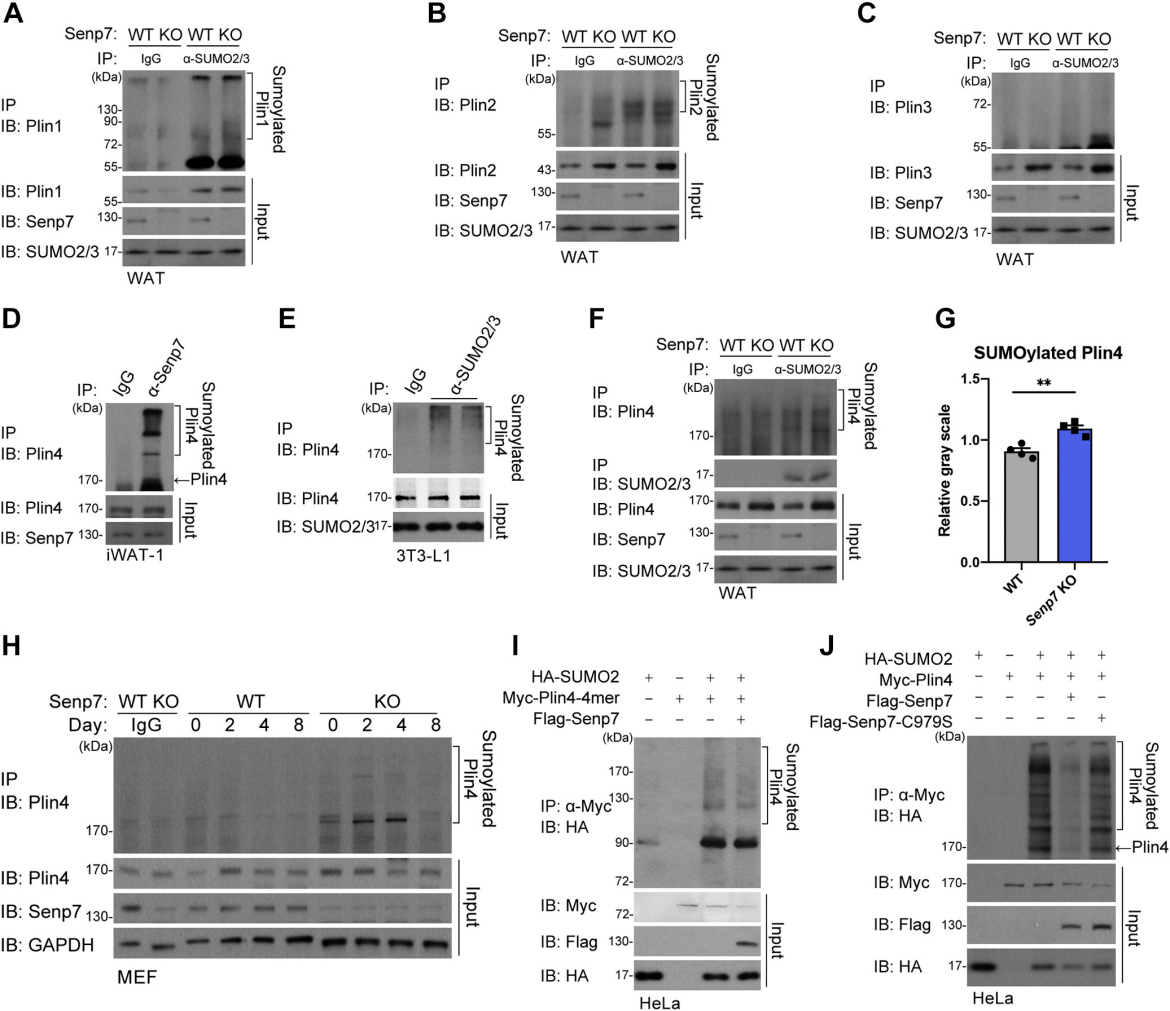
**Plin4 is deSUMOylated by Senp7.***A*–*C*, SUMO2/3-conjugated proteins in WT and *Senp7* KO mice WAT were immunoprecipitated (IP) with anti-SUMO2+3 antibody, and SUMO-Plin1/2/3 proteins were blotted with anti-Plin1 (*A*), anti-Plin2 (*B*), and anti-Plin3 (*C*). Cell lysate was immunoblotted with anti-Senp7, anti-SUMO2+3, and anti-Plin1/2/3 antibody as input. The *third* and *fourth panels* in *A* were reused in *B*, *C*, and *F*. *D*, Senp7-interacted proteins in iWAT-1 cells were IP with anti-Senp7 antibody, and SUMO-Plin4 proteins were blotted with anti-Plin4. Cell lysate was immunoblotted (IB) with anti-Senp7 and anti-Plin4 antibody. *E*, SUMO2/3-conjugated proteins in 3T3-L1 cells were IP with anti-SUMO2+3 antibody, and SUMO-Plin4 proteins were blotted with anti-Plin4. Cell lysate was immunoblotted with anti-SUMO2+3 and anti-Plin4 antibody. *F*, SUMO2/3-conjugated proteins in WT and *Senp7* KO mice WAT were IP with anti-SUMO2+3 antibody, and SUMO-Plin4 proteins were blotted with anti-Plin4. Cell lysate was immunoblotted with anti-Senp7, anti-SUMO2+3 and anti-Plin4 antibody. *G*, *gray scale values* of SUMO2/3-conjugated Plin4 was analyzed from (*F*) and other three independent experiments. Values were normalized to *gray scale values* of IP productions that were immunoblotted with anti-SUMO2+3 antibody. Mean ± SD; ∗∗*p* < 0.01 by two-tailed *t* test. *H*, WT and *Senp7* KO MEFs were differentiated with adipogenic stimuli. SUMO2/3-conjugated proteins in MEFs on day 0, 2, 4, and 8 were IP with anti-SUMO2+3 antibody, and SUMO-Plin4 proteins were blotted with anti-Plin4. Cell lysate was immunoblotted with anti-SUMO2+3 and anti-Plin4 antibody. *I*, HeLa cells were transfected with HA-SUMO1, Myc-Plin4-4mer, 3xFlag-Senp7 as indicated. Myc-Plin4-4mer proteins were pulled down by anti-Myc beads from these cell lysates. Bound proteins were blotted with anti-YPYDVPDYA (HA) (*top panel*). Cell lysate was immunoblotted with anti-Myc antibody (*second panel*), anti-Flag antibody (*third panel*), or anti-HA antibody (*fourth panel*). *J*, HeLa cells were transfected with HA-SUMO1, Myc-Plin4, 3xFlag-Senp7, and 3xFlag-Senp7-C979S as indicated. Myc-Plin4 proteins were pulled down by anti-Myc beads from these cell lysates. Bound proteins were blotted with anti-HA (*top panel*). Cell lysate was immunoblotted with anti-Myc antibody (*second panel*), anti-Flag antibody (*third panel*), or anti-HA antibody (*fourth panel*). All experiments were performed at least twice. iWAT, inguinal white adipose tissue; LD, lipid droplet; MEF, mouse embryonic fibroblast; Plin, perilipin; SENP, sentrin/SUMO-specific protease; SUMO, small ubiquitin-like modifier.

To elucidate the mechanisms by which Senp7 regulates the size of LDs, we conducted an immunoprecitation (IP) assay, followed by mass spectrometry (MS) analysis to identify proteins that interact with Senp7. The result showed that Plin4, a lipid-coated protein, was enriched (Table S1). Although we identified multiple peptides derived from other Plins, including Plin1 and Plin3, we did not observe a definite interaction between Senp7 and Plin1/2/3 in immortalized preadipocytes cell lines from WAT (iWAT-1) cells by IP (Fig. S5, *A*–*C*). In addition, *Senp7* deficiency did not affect the SUMOylation level of Plin1 or Plin2 in WAT (Fig. 5, *A* and *B*). Furthermore, Plin3 could not be SUMOylated with SUMO2/3 in WAT (Fig. 5*C*). Our results demonstrate that Plin1, 2, or 3 are not the substrates for Senp7.

Next, we confirmed that SUMO2/3-conjugated Plin4 interacted with Senp7 by IP (Fig. 5, *D* and *E*). This result is consistent with a previous study, suggesting that Plin4 is modified with SUMO2 (34). Furthermore, a significant increase of SUMO2/3-conjugated Plin4 was observed in the WAT of *Senp7* KO mice, suggesting that *Senp7* deficiency leads to the accumulation of SUMO2/3-modified Plin4 (Fig. 5, *F* and *G*). As endogenous sumoylome in adipocytes is dynamic during lipogenesis (35), we investigated the SUMO2/3-modification of Plin4 at different time points of lipogenesis. SUMOylated Plin4 decreased in WT mouse embryonic fibroblasts after 2 days of lipogenic induction (Fig. 5*H*). In contrast, *Senp7* KO mouse embryonic fibroblasts showed accumulated SUMO2/3-conjugated Plin4 (Fig. 5*H*). Consistently, we observed an increase of SUMO2/3-conjugated Plin4 in *Senp7* KO iWAT-1 cells (Fig. S5*D*). These data suggest that Senp7 deSUMOylates Plin4 during lipogenesis.

To explore the deSUMOylase activity of Senp7, we generated plin4-overexpressed cells. Human PLIN4 contains amphipathic helices (AHs) domain, which anchors PLIN4 to the surface of LDs (36). The reported SUMO-modified lysine residues (34) were contained within the predicted AHs of mice Plin4 protein, which repeats in a highly homologous format 33 times (Fig. S5, *E*–*G*). As it is difficult to synthesize and sequence the full-length *Plin4*, we first constructed an artificial *Plin4* containing four AHs with SUMOylation sites, referred as Plin4-4mer (Fig. S5*E*). We expressed Plin4-4mer in HeLa cells and confirmed that the SUMOylation of Plin4-4mer was decreased while overexpressing *Senp7* (Fig. 5*I*). Moreover, we cloned full-length *Plin4* from the mouse genome using the Red/ET recombination system. Senp7 could deconjugate the SUMO2 modification chain from Plin4, whereas Senp7 catalytically deficient mutant, Senp7-C979S (11) (Fig. S5*H*), could not (Fig. 5*J*). These data demonstrate that Senp7 was able to deSUMOylate the SUMO2/3 conjugations from Plin4.

### *Senp7* deficiency decreases the LD-coated Plin4

**Figure 6:**
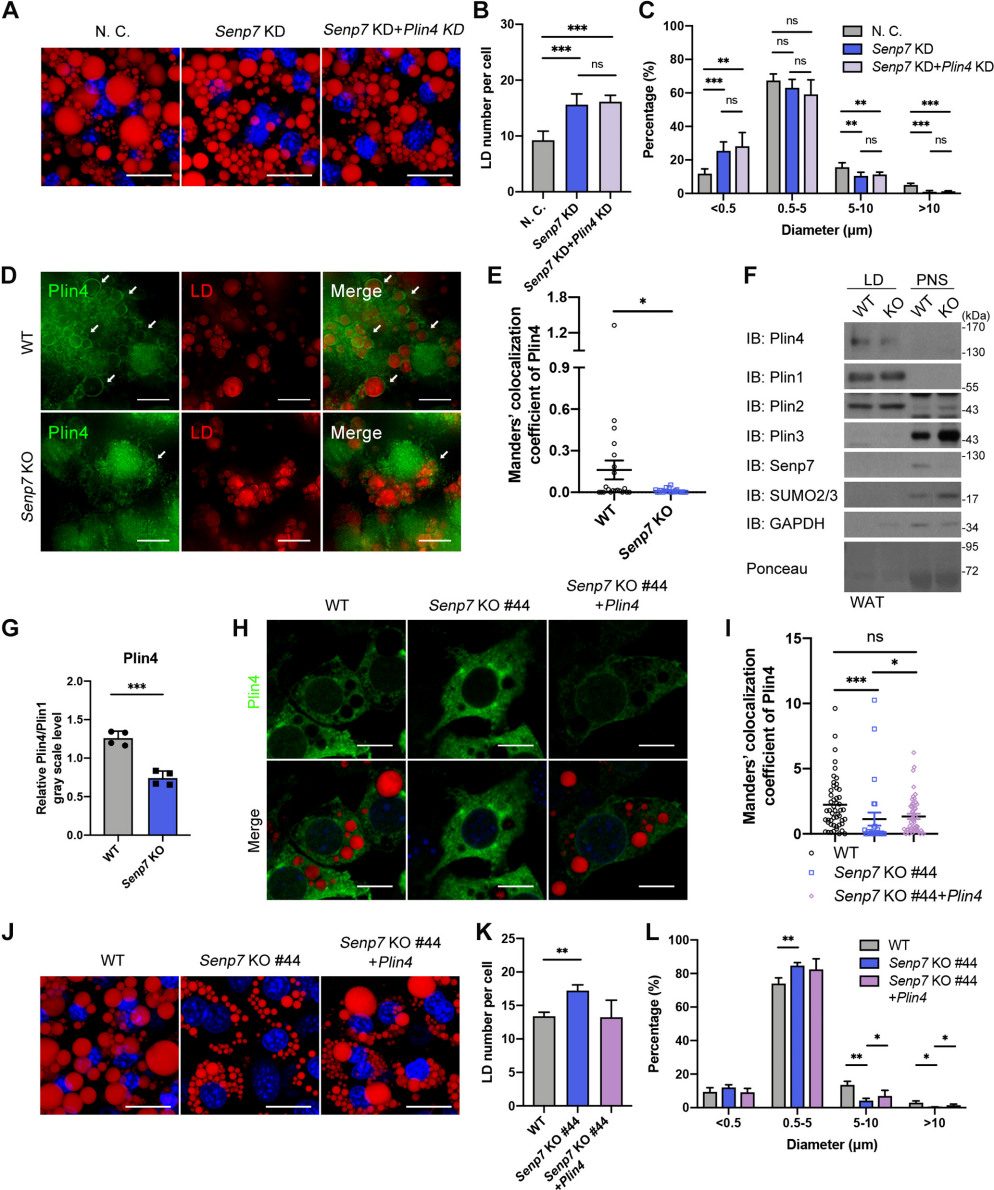
***Senp7* deficiency decreases the LD-binding Plin4.***A*–*C*, *Senp7* and *Plin4* in iWAT-1 cells were knocked down as indicated. The cells were differentiated for 8 days. LDs were stained with Nile red (*red*), and the diameters of LDs in 100 cells for each independent experiment were quantified (n = 3). The scale bars represent 20 μm. Mean ± SD; ∗∗*p* < 0.01, ∗∗∗*p* < 0.001; and ns, no significance by one-way ANOVA with the Tukey post hoc test. *D*, colocalization of LDs (*red*) and Plin4 (*green*) in differentiated WT or *Senp7* KO primary preadipocytes on day 8. Images were captured by GE DeltaVision OMX super-resolution microscope. The scale bars represent 10 μm. *E*, quantification of Plin4-LD colocalization. The intensity of Plin4-LD colocalization was automatically analyzed by Imaris. Manders’ co-occurrence analysis of Plin4 and LD. The scale bars represent10 μm. Z-stack interval, 1.5 μm. n = 20 cells; mean ± SEM; ∗*p* < 0.05 by Mann–Whitney test. *F*, LD fractions were isolated from iWAT derived from WT and *Senp7* KO mice. LD proteins were immunoblotted with anti-Plin4, anti-Plin1, anti-Plin2, anti-Plin3, anti-Senp7, anti-SUMO2/3, or anti-GAPDH antibody. One representative blot from three independent experiments is shown. *G*, the *gray scale values* of Plin4 from four independent experiments were measured and were normalized to the value of Plin1. The value of *gray scale* was analyzed by Image J (https://imagej.net/software/imagej/). Mean ± SD; ∗∗∗*p* < 0.001; ns, no significance by two-tailed *t* test. *H* and *I*, *Senp7* KO iWAT-1 cells were transfected with Plin4-expressed plasmid and were differentiated. Images of LDs (*red*), Plin4 (*green*), and nucleus (*blue*) on day 4. Images were captured by ZEISS confocal microscope. The intensity of Plin4-LD colocalization was automatically analyzed by Imaris and was normalized to the total intensity of Plin4 in each cell. WT = 50 cells, #44 = 26 cells, #44+Plin4 = 50 cells. The scale bars represent 10 μm. Z-stack interval, 1.5 μm. Mean ± SEM; ∗*p* < 0.05, ∗∗∗*p* < 0.001; and ns, no significance by Kruskal–Wallis test with Dunn’s post hoc test. *J*–*L*, *Senp7* KO #44 iWAT-1 cells were transfected with Plin4 by electroporation and were differentiated for 8 days. LDs were stained with Nile red (*red*), and the diameters of LDs in 50 cells for each independent experiment were quantified (n = 3). The scale bars represent 20 μm. Mean ± SD; ∗∗*p* < 0.01, ∗∗∗*p* < 0.001; and ns, no significance by one-way ANOVA with the Tukey post hoc test. All experiments were performed at least twice. iWAT, inguinal white adipose tissue; LD, lipid droplet; Plin, perilipin; SUMO, small ubiquitin-like modifier.

As a posttranslational modification, SUMOylation can induce SUMO-dependent ubiquitination, thus leading to the degradation of its substrates (37). However, Plin4 expression was elevated in *Senp7* KO tissues and cells (Figs. 5*F* and S6, *A*–*E*), suggesting that Senp7 did not decrease the levels of Plin4 by SUMO-dependent degradation. To determine if the increase of Plin4 causes irregular LD morphology, we knocked down *Plin4* in *Senp7*-deficient iWAT-1 cells (Fig. S6, *F* and *G*). The LD number and the size distribution pattern were not rescued by downregulating Plin4 (Fig. 6, *A*–*C*). These results indicate that *Senp7* deficiency did not impair LD size through increasing the expression of Plin4. The upregulated expression of Plin4 may be a compensatory response to the dysfunction of SUMOylated Plin4.

Plin4 is typically found in the cytosol in preadipocytes and is then mobilized to the surface of nascent LDs to package newly synthesized TG during lipogenesis (27). Therefore, we investigated whether Senp7 could regulate the translocation of Plin4 during lipogenesis. On day 4 postdifferentiation, Plin4 was observed coating LDs in WT primary adipocytes (Fig. 6, *D* and *E*). A significantly decreased level of LD-coated Plin4 was observed in *Senp7* KO cells (Fig. 6, *D* and *E*). We further confirmed that Plin4 was enriched in the LD fraction of WT adipose tissue but was significantly reduced in *Senp7* KO adipose tissue (Figs. 6, *F* and *G* and S6, *H*–*J*), suggesting that Senp7 deficiency decreases LD-coated Plin4. We hypothesized that the altered LD morphology was caused by the decreased LD-coated Plin4 in *Senp7*-deficient cells. To restore LD-coated Plin4, we overexpressed *Plin4* in *Senp7* KO iWAT-1 cells to upregulate unmodified Plin4 (Fig. S6, *K* and *L*). Both unmodified Plin4 and SUMO2/3-conjugated Plin4 increased (Fig. S6*L*), and the LD-coated Plin4 was partially restored (Fig. 6, *H* and *I*). On day 8, there were more LDs exceeding 10 μm in *Plin4*-overexpressed cells compared to *Senp7* KO cells (Fig. 6, *J* and *L*). However, the overexpression of Plin4 could not rescue the total number of LDs in cells, possibly because of the increased amount of SUMOylated Plin4 (Figs. 6*K* and S6*L*). These data demonstrate that the lack of Senp7 could decrease the LD size by diminishing the LD-coated Plin4.

**Figure 7:**
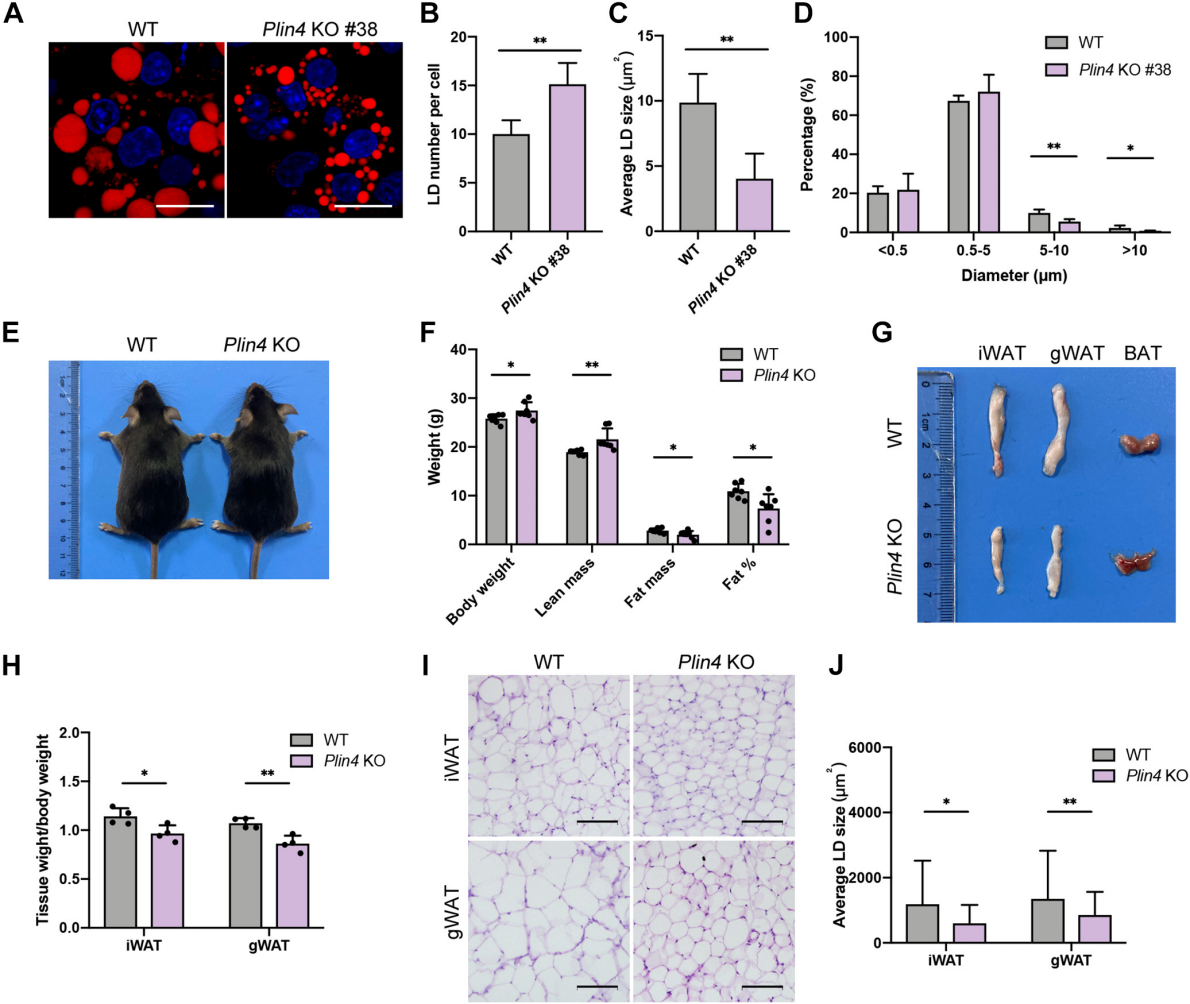
***Plin4* KO mice show less fat accumulation in white adipose tissues.***A*–*D*, Nile red staining of differentiated iWAT-1 on day 8 and quantification of diameter and number of the LDs in 50 cells for each independent experiment (n = 3). The scale bars represent 20 μm. Mean ± SD; ∗*p* < 0.05, ∗∗*p* < 0.01; and ns, no significance by two-tailed *t* test. *E*, gross morphology of 10-week-old male mice. *F*, body composition of female WT and *Plin4* KO mice measured by DEXA after 8 weeks on a regular chow diet. n = 7; mean ± SD; ∗*p* < 0.05∗∗*p* < 0.01 by two-tailed *t* test. *G* and *H*, dissection image (*G*) and weight normalized to body weight of organs (iWAT, gWAT, BAT) (*H*) of 8-week-old female WT and *Plin4* KO mice. n = 4; mean ± SD; ∗*p* < 0.05, ∗∗*p* < 0.01 by two-tailed *t* test. *I*, H&E staining of iWAT and gWAT sections from 14-week-old female WT and *Plin4* KO mice. The scale bars represent 100 μm. *J*, statistics of adipocyte size from iWAT and gWAT of 14-week-old female WT and *Plin4* KO mice. Three slices from each tissue with three mice per genotype were analyzed. n = 1800 cells; mean ± SD; ∗∗∗*p* < 0.001 by Mann–Whitney test. Error bars are represented as mean ± SD. ∗*p* < 0.05, ∗∗*p* < 0.01. BAT, brown adipose tissue; gWAT, gonadal white adipose tissue; iWAT, inguinal white adipose tissue; LD, lipid droplet; Plin, perilipin.

To further confirm the role of Plin4 in lipid accumulation, we generated *Plin4* KO iWAT-1 cells and induced the cells with lipogenic stimuli. At 8 days post induction, *Plin4* KO iWAT-1 cells harbored more but smaller LDs and exhibited a reduced number of mature LDs (>10 μm) (Fig. 7, *A*–*D*). Moreover, we examined the body composition of *Plin4* conventional KO (*Plin4* KO) mice. Eight-week old male *Plin4* KO mice showed normal morphology but displayed a slightly higher body weight (Fig. 7, *E* and *F*). Compared to WT mice, *Plin4* KO mice contained decreased fat mass with a visible reduction in iWAT and gWAT depots (Fig. 7, *F*–*H*). Accordingly, smaller adipocytes were observed in both iWAT and gWAT (Fig. 7, *I* and *J*). These data indicate that loss of Plin4 in mice led to reduced fat mass and smaller adipotyces, which is similar to that observed in *Senp7* KO and AKO mice. Taken together, our data suggest that Plin4 was involved in the LD enlargement under the regulation of Senp7.

### Senp7 regulates the LDs size through deSUMOylating Plin4

**Figure 8:**
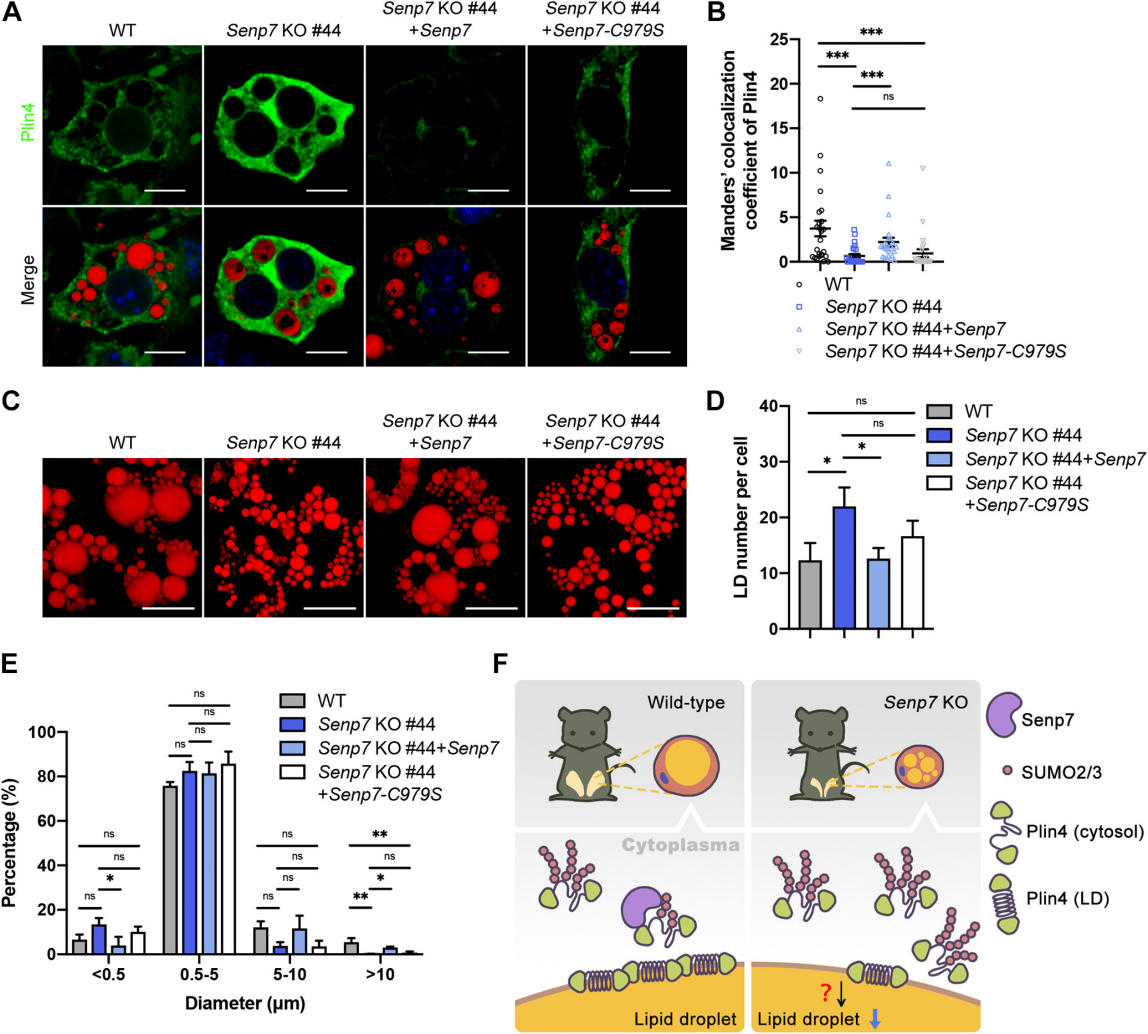
**Senp7 regulates the LDs size through deSUMOylating Plin4.***A* and *B*, *Senp7* KO #44 iWAT-1 cells with stable expression of Senp7 or Senp7-C979S protein was differentiated. Images of LDs (*red*), Plin4 (*green*), and nucleus (*blue*). Images were captured by ZEISS confocal microscope. The intensity of Plin4-LD colocalization was automatically analyzed by Imaris and was normalized to the total intensity of Plin4 in each cell. n = 25 cells. The scale bars represent 10 μm. Z-stack interval, 1.5 μm. Mean ± SEM; ∗∗*p* < 0.01, ∗∗∗*p* < 0.001; and ns, no significance by Kruskal–Wallis test with Dunn’s post hoc test. *C*–*E*, *Senp7* KO iWAT-1 cells (*Senp7* KO #44) with stable expression of Senp7 or Senp7-C979S protein were differentiated and stained with Nile red on day 8, and the diameters of LDs in 30 cells for each independent experiment were quantified (n = 3). The scale bars represent 20 μm. Mean ± SD; ∗*p* < 0.05, ∗∗*p* < 0.01; and ns, no significance by one-way ANOVA with the Tukey post hoc test. *F*, model graph proposing the role of Senp7 in LD maturation by regulating LD-coated Plin4 in a SUMO-dependent manner. All experiments were performed at least twice. iWAT, inguinal white adipose tissue; LD, lipid droplet; Plin, perilipin; SENP, sentrin/SUMO-specific protease; SUMO, small ubiquitin-like modifier.

To confirm that the regulatory effect of Senp7 on Plin4 is dependent on its catalytic activity, we expressed *Senp7-C979S* in *Senp7* KO iWAT-1 cells (Fig. S6*M*). The re-expression of *Senp7* in *Senp7* KO iWAT-1 increased the LD-localized Plin4, but this effect was not observed in *Senp7-C979S* group (Fig. 8, *A* and *B*). Moreover, the total number of LD and the number of mature LD (>10 μm) were recovered by the expression of WT *Senp7* but not *Senp7-C979S* in *Senp7* KO cells (Fig. 8, *C*–*E*). Overall, these data suggest that Senp7 regulates the morphology of LDs by modulating Plin4 in a SUMOylation-dependent manner (Fig. 8*F*).

## Discussion

In this study, we identified Senp7 as a novel regulator of adipose lipid storage. Loss of *Senp7* caused defects in lipid accumulation *in vivo*, leading to a decrease in fat pad mass. The defects were directly caused by *Senp7* deficiency in adipose tissues. *Senp7* deficiency could lead to an altered LD size distribution pattern characterized by the increased number and smaller size of LDs in differentiated adipocytes. Mechanistically, Senp7 could deSUMOylate Plin4 and promote its localization to LD, suggesting a potential role of Senp7 in LD maturation during lipid storage (Fig. 8*F*).

*Senp7*-deficient mice displayed a lack of WAT, which is closely related to lipodystrophy. Patients with lipodystrophy are characterized by a generalized or partial absence of adipose tissue, accompanying with insulin resistance, dyslipidemia, and fatty liver (38, 39). Common mice models of inherited lipodystrophy revealed grossly visible fat reductions of 80% (40, 41, 42), even presenting with virtually no WAT or BAT (43, 44, 45, 46). Also, those mouse models showed disordered serum glucose and liver steatosis (40, 41, 42, 43). In this study, male and female *Senp7* KO mice manifested a reduction of 33.82% and 21.00% in fat mass, respectively. Male and female *Senp7* AKO mice manifested a reduction of 17.16% and 14.74% in fat mass, respectively. However, *Senp7* KO and AKO mice did not develop insulin resistance or fatty liver. This might be related to the relatively mild WAT loss in *Senp7*-deficient mice, which is consistent with the clinical characteristics of asymptomatic partial lipodystrophy patients (39).

Female *Senp7* KO and AKO mice showed higher respiratory exchange ratio (RER). However, oxygen consumption and carbon dioxide production were unaffected between the genotypes. Mice with higher RER utilize carbohydrate as the primary energy substrate rather than fatty acid (47). This might be an adaptation to the altered lipid availability due to the loss of adipose tissue, which leads to a greater use of carbohydrates.

Our data demonstrate that the impaired adipocyte enlargement is owing to the smaller size of LDs in *Senp7*-deficient adipocytes. The LD life cycle in mammalian cells starts with the biogenesis and early growth phase, and then it selectively grows and maintains as mature LD (48). Each of the steps would diversify LD morphology (49, 50, 51). LDs can grow through the fusion of two LDs (49), however, fluorescence recovery after photobleaching analysis revealed similar lipid exchange rates in LDs derived from *Senp7*-deficient adipocytes, suggesting that Senp7 does not attenuate lipid mobility after LDs attach to each other (Fig. S7, *A* and *B*). We will further investigate whether Senp7 plays a role in the LD–LD contact rate. Additionally, the bilayer phospholipid composition is a key parameter of LD budding and maintaining (52). We will further uncover whether Senp7 could regulate the phospholipid composition of LD membrane to manipulate LD size pattern.

We found that Senp7 regulated LD size *via* deSUMOylation of Plin4. A SNP at *PLIN4* locus was reported, and its variant alleles were associated with lower obesity risk in women (53). Moreover, *Plin4* conventional KO mice displayed a significant reduction in fat mass (Fig. 7*F*). Meanwhile, *Plin4* KO iWAT-1 cells showed an impaired LD pattern (Fig. 7, *A*–*D*), supporting that Plin4 plays a role in LD metabolism.

Senp7 could interact with Plin4 and regulate the subcellular localization of Plin4. Plin4, as reported, distributes throughout the cytosol and could coat smaller, peripheral droplets (27). This constitutes a prepared reservoir of lipid-coated proteins for rapid packaging of newly synthesized TG and to enhance energy storage during nutrient excess (27). Consistently, we observed that Plin4 coated the surface of tiny LDs, as well as the localization of LDs of various sizes, providing insight into the potential role of Plin4 during the maturation of LDs. Yet, the mechanism by which Plin4 translocates between the cytosol and the surface of the LDs is still unclear. Plin4 targets LDs through AHs adsorbing at polar-apolar interfaces to directly interact with the LD lipid surface (36). Plin4 AH region contains SUMO-conjugated sites (34). We found elevated Plin4 expression in *Senp7*-deficient cells and tissues with accumulated SUMO2/3-modified Plin4 (Figs. S6, *A*–*E* and 5*F*). We hypothesized that SUMOylation in this region might influence polar and charged residues, thereby interrupting the direct LD interaction. Hence, when unmodified Plin4 was overexpressed, it was found to partially rescue its location on LD surface and facilitate LD growth (Fig. 6, *H*–*L*). For further study, we will mutate SUMO-conjugated sites in Plin4 to investigate whether SUMOylation could regulate the subcellular localization of Plin4.

Taken together, the deficiency of *Senp7* in mice impairs fat accumulation and LD expansion in adipocytes. Identifying new factors that enable healthy adipose tissue expansion brings critical implications for treating lipid-associated diseases, including obesity and lipodystrophy.

## Experimental procedures

### Mice

B6/N-Senp7^tm1b^Nju mice were provided by the Nanjing Biomedical Research Institute of Nanjing University (strain NO. XM000389). LoxP sites were inserted surrounding the Senp7 gene exons 4. B6/N-Senp7^tm1b^Nju mice were mated with FLPO mice (purchase from GemPharmatech Co, Ltd, strain NO.T002183) carrying the Flippase that could delete LacZ element in KO first mice model to generate Senp7^fl/fl^ mice. Then Senp7^fl/fl^ mice were mated with lines carrying the Cre recombinase driven by the ACTB (gift from Wellcome Sanger Institute), or the Adiponectin (gift from Dr Zhengji Gan; Nanjing University). *Senp7*^fl/fl^ and *Senp7* AKO mice were fed a HFD containing 60% energy from fat (Research Diets) at 8 weeks of age for 12 weeks. Mice were housed in a specific pathogen-free and Association for Assessment and Accreditation of Laboratory Animal Care International-accredited animal facility. Animal welfare and experimental procedures were approved by the Institutional Animal Care and Use Committee of the Model Animal Research Center, Nanjing University.

### Metabolic characterization

For body composition measurements, body weight was measured and mice were anesthetized with 1% ketamine (1 mg/10 g body weight) and 0.05% xylazine (0.05 mg/10 g body weight). Body composition was examined by a DEXA system (PIXImus 2, GE lunar).

For metabolic characterization, mice were maintained on chow diet and housed in a computer-controlled open-circuit monitoring system (Oxymax indirect calorimetry system, Oxymax/CLAMS, Columbus Instruments). The mice were kept at least 1 day of acclimation and 3 days of monitoring. Parameters including O_2_ consumption, CO_2_ production, and daily food intake were detected, and locomotor activities were measured on the *x*-axis through infrared beams. RER and heat production levels were calculated from the O_2_ consumption, CO_2_ production, and daily food intake values.

### TG and NEFA measurement

Serum TG content was quantified by LabAssay TG (FUJIFILM Wako Chemicals U.S.A. Corporation, 290-63701). Serum nonestesterified fatty acid (NEFA) was quantified by LabAssay NEFA (FUJIFILM Wako Chemicals U.S.A. Corporation, 633-52001).

Cells containing LDs were washed with PBS and lysed in radio immunoprecipitation assay lysis buffer (50 mM Tris–HCl, pH 7.4, 150 mM NaCl, 1% Nonidet P-40, 0.1 mM EDTA, 1 mM DTT). After centrifugation at 1000*g* for 5 min, the upper lysate was collected. TG level was measured using LabAssay TG and normalized per microgram of protein.

### Histology

Four percent paraformaldehyde-fixed, paraffin-embedded adipose and liver tissue were sectioned at 10-μm intervals for adipose tissues and at 5-μm intervals for liver. The sections were stained with H&E. Images were captured using an Olympus BX53 microscope.

### Cell culture

HeLa cells were cultured in high-glucose Dulbecco’s modified Eagle medium (DMEM, Hyclone, SH30022.01) containing 10% fetal bovine serum (FBS, PAN-Biotech, ST30-3302), 2 mM l-glutamine, 100 U/ml penicillin, and 100 μg/ml streptomycin at 37 °C in a humidified incubator containing 5% CO_2_.

3T3-L1 cells were cultured in DMEM (Gibco, 11995065) containing 10% newborn calf serum (NCS, Sigma, 13063C), 100 U/ml penicillin, and 100 μg/ml streptomycin at 37 °C in a humidified incubator containing 5% CO_2_.

Immortalized preadipocytes cell lines from WAT (iWAT-1 cells, gift from Dr Xinran Ma; East China Normal University) were cultured in high-glucose DMEM (Gibco) containing 10% FBS (Gibco, 10099141C), 2 mM l-glutamine, 100 U/ml penicillin, and 100 μg/ml streptomycin at 37 °C in a humidified incubator containing 5% CO_2_. For adipogenic differentiation, 2-day postconfluent cells (day 0) was induced by the addition of insulin (5 μg/ml, Sigma, I9278), dexamethasone (1 μM, Sigma, D4902), isobutyl-1-methylxanthine (0.5 mM, Sigma, I5879), and rosiglitazone (1 μM, Sigma, R2408). On day 2, the medium was replaced with the basic medium containing 5 μg/ml insulin. This medium was changed every 2 days until the end of differentiation.

For primary preadipocytes, subcutaneous fat tissues were harvested from 14-day-old mice and cut into small pieces with scissors, following incubation in adipose isolation buffer containing 1 mg/ml collagenase type I (Worthington Biochemical, LS004196) for 25 min at 37 °C with gentle shaking. Cells were then filtered through cell strainer (40 μm). Preadipocytes were collected as a pellet by centrifugation at 250*g* for 5 min at 4 °C. Remove supernatant and resuspend pellet in 1 ml basic medium (DMEM, 10% FBS, 1% penicillin/streptomycin, 50 μg/ml gentamicin, 25 μg/ml sodium ascorbate). For adipogenic differentiation, 2-day -postconfluent cells (day 0) was induced by the addition of insulin, dexamethasone, isobutyl-1-methylxanthine, and rosiglitazone. On day 2, the medium was replaced with the basic medium containing 1 μg/ml insulin and 1 mM rosiglitazone. The medium was renewed every 2 days with basic medium since day 4. The differentiated cells were then stained with Oil Red O. Briefly, cells were washed with PBS, fixed with 4% paraformaldehyde for 30 min, and stained with Oil Red O for 15 min using a 3:2 (v/v) dilution in double-diluted H_2_O of a 0.5% stock solution (in isopropanol). Cells were then washed once with 60% isopropanol and twice with PBS.

### Generation of KO cell line using CRISPR/Cas9

Generation of KO cell lines was performed according to previous studies (54). In brief, single-guide RNAs (sgRNAs) were designed using tefor (https://http://crispor.tefor.net/) and constructed into pX459 vector. Plasmids were electroporatly transfected into iWAT-1 cells. Cells were then cultured for 24 h and treated with 1 μg/ml of puromycin for 3 days. The drug-resistant cells were then diluted and plated on 96-well plates to form a single colony. Each colony was genotyped and the knockdown efficiency was evaluated by qRT-PCR analysis and Western blot analysis. sgRNAs used for knocking out *Senp7*: sequence 1: 5′-TTTGTAGAGATTGTTACAGA; sequence 2: 5′-TGAGACTAGCACAATGGTAT. sgRNAs used for knocking out *Plin4*: sequence 1: 5′-CTTCAGCTCTGCCCGGAACC; sequence 2: 5′-TGTTTGTAAGTCCTTTGTGG.

### Quantitative real-time PCR

Total RNA was extracted from mouse tissues with RNAiso Plus (TAKARA). Reverse transcription was performed by a standard procedure (Vazyme, R323-V10.1) using 1 mg of total RNA. qRT-PCR was performed with ChamQTM SYBR qPCR Master Mix (Vazyme, Q311-V9.1). Relative standard real-time PCR was performed on the Roche Light Cycler instrument. Relative gene expression was calculated by the hyperbolic method and was normalized to the internal control gene *Rplp0* (alias *36B4*). *Rplp0* forward: 5′-GCAGACAACGTGGGCTCCAAGCAGAT; *Rplp0* reverse: 5′-GGTCCTCCTTGGTGAACACGAAGCCC. *Senp7* forward: 5′-ACACCCAGAGTTATACTGACGG; *Senp7* reverse: 5′-TGGTTTGGGACCACTTTCAGATA. *Plin4* forward: 5′-GTGTCCACCAACTCACAGATG; *Plin4* reverse: 5′-GGACCATTCCTTTTGCAGCAT.

### Plasmid construct and gene overexpression

Expression plasmid for Flag-tagged Senp7 was generated by inserting mouse Senp7 into pEGFP-N1. Point mutations of Senp7 were introduced by a PCR-based site-directed mutagenesis.

Expression plasmid for Plin4-4mer was generated by cloning sequences encoding amino acids 1 to 229 and 1187 to 1649 into pEGFP-N1-Myc vector.

Expression plasmid for full-length Plin4 was generated by Red/ET strategy. Briefly, truncations of Plin4 encoding amino acids 1 to 206 and 1202 to 1649 were amplified by PCR from mouse complementary DNA and were then cloned into pEGFP-N1-Myc vector (referred as pEGFP-Myc-Plin4-truncation). pSC101-Red/ET-containing BAC clone RP23-233H19 was supplied with 3.5% L-Arabinose and were incubated at 37 °C for 1 h, shaking at 220 rpm to induce the expression of genes mediating Red/ET system. Then linearized pEGFP-Myc-Plin4-truncation was introduced into pSC101-Red/ET–expressing RP23-233H19 using the Gene Pluser XCell Electroporator (Bio-Rad), following protocols provided by the manufacturer.

HeLa cells were transfected by Lipofectamine 2000, following protocols provided by the manufacturer. At 48 h post transfection, cells were collected for protein analysis. iWAT-1 cells and primary preadipocytes were transfected using the Neon transfection system (Invitrogen, MPK5000), following protocols provided by the manufacturer. Electroporation parameters were 1650 V, 20 ms, once. Cells were then plated on 12-well plates (1 × 10^5^ cells/well) and treated with the differentiation mixture for later analysis.

### RNA interference

siRNAs were introduced into iWAT-1 preadipocytes by electroporation. Electroporation parameters were 1150 v, 20 ms, thrice. Cells were then cultured for 48 h and harvested. The knockdown efficiency for each protein was evaluated by qRT-PCR analysis. Senp7-mus: 5′-GCCAUGUAAUAAAGACCAATT. Plin4-mus: 5′-GCAUUUACAGAGCCACUAATT.

### Antibodies and regents

The antibody against Senp7 (LS-C98791) was purchased from LSBio, the antibody against SUMO2/3 (ab81371) and Plin1 (ab172907) were purchased from ABCam, the antibody against Plin2 (15294-1-ap) was purchased from Proteintech, the antibody against Plin3 (NB110-40764SS) was purchased from NOVUS, the antibody against Plin4 (ABS526), PLIN4 (HPA044682), and Flag (F1804) were purchased from Sigma, the antibody against Myc (#2276) and HA (#3724) were purchased from CST, and the antibody against α-tubulin (BS1699) was purchased from Bioworld Technology, Inc. All the primary antibodies were diluted in a ratio of 1:1000. Secondary antibodies, anti-mouse IgG and anti-rabbit IgG, were purchased from Sigma-Aldrich. All the secondary antibodies were diluted in a ratio of 1:10,000.

### Western blotting

Tissue and whole-cell lysates were prepared on ice in radio immunoprecipitation assay supplemented with protease inhibitors, including PMSF, Na_3_VO_4_ and NaF, plus cocktail protein inhibitor. The concentration of protein samples was determined by the Bradford bioassay-Bradford protein assay kit (Sangon). Protein samples were electrophoresed in suitable SDS-PAGE gels and transferred in low temperature to polyvinylidene difluoride membranes (Amersham Bioscience). Fat-free milk (5%) was used to block blots at room temperature for 1 h, and then the blots were incubated with primary antibody overnight at 4 °C. After being washed with tris-buffered saline and 0.5% Tween-20, the blots were incubated with corresponding secondary antibody for 1 h at room temperature. Immunoreactive bands were visualized by chemiluminescence.

### Immunoprecipitation

Cells were collected 48 h after transfection and lysed in IP lysis buffer (20 mM Tris–HCl pH 8, 137 mM NaCl, 1% Nonidet P-40, 2 mM EDTA), supplemented with protease inhibitor cocktail (Sigma, P8340) and 1 mM N-ethylmaleimide (deSUMOylase inhibitor, Sigma, 4259). After being centrifuged at 17,000*g* for 20 min at 4 °C, the supernatants were collected and were precleaned with 20 μl protein G Sepharose beads (CYTIVA, 17-0618-01). For pulldown assay, the cell lysates were incubated with indicated antibodies (1 μg/mg) at 4 °C overnight and were then added with protein G Sepharose beads for 4 h at 4 °C; for IP assay, the cell lysates were added with anti-c-Myc beads (Thermo Fisher Scientific, 20168) or anti-Flag beads (Sigma, A2220) and were rotated at 4 °C overnight. Next, the beads were washed five times with tris-buffered saline and 0.5% Tween-20 buffer. The immunoprecipitates were treated with 30 μl of 2% SDS solution containing 1 μM DTT and analyzed by Western blotting.

### MS analysis

Mouse primary preadipocytes were differentiated for 2 days. Cell lysate was incubated with anti-Senp7 beads or mouse IgG individually. Then proteins were eluted and collected followed by SDS-PAGE. Proteins were digested into peptides by trypsin. Tryptic peptides were desalinated and then subjected to MS analysis. Mass spectral analysis was performed on the AB Sciex Triple TOF 5600+ mass spectrometer (AB Sciex) with an electrospray ionization probe operated in positive ion mode. The raw data were processed using AB SCIEX ProteinPlot software (version 4.5, https://sciex.com/products/software/proteinpilot-software) in its standard mode. Data were searched against the February 2018 UniProt mouse database (61,314 entries). Peptides with confidence >95% were considered for further analysis. Three trypsin missed cleavages were considered. The minimum length of peptides was seven amino acids. Identified proteins in IgG control sample were excluded from protein result of anti-Senp7 pulldown sample. The analysis results were listed in Table S1.

### Immunofluorescence

For immunostaining, cells cultured on coverslips were washed twice with PBS, fixed with 4% paraformaldehyde for 20 min, and were blocked with immunofluorescence blocking buffer (5% bovine serum albumin (BSA) in PBS supplemented with 0.3% Triton X-100 and 0.1% sodium deoxycholate) for 1 h and incubated with primary antibodies (1:100 diluted) in PBS supplemented with 0.1% BSA, 0.3% Triton X-100, and 0.1% sodium deoxycholate overnight at 4 °C and then with secondary antibodies (1:200 diluted) for 1 h at room temperature. LDs were stained with Nile red (MCE, HY-D0718) in PBS for another 20 min. Images were captured using a ZEISS LSM 880 microscope or a GE DeltaVision OMX super-resolution microscope.

### Image processing

Adipocyte size was measured automatically using Cell Profiler software (version 4.2.5, https://cellprofiler.org/) as described (55). Images of H&E-stained WAT sections were acquired and were analyzed by Cell Profiler following the pipeline: http://cellprofiler.org/forum/viewtopic.php?f.14&t.1687&hilit.adipocyte&start.15.

LDs were sized automatically using Image-Pro Plus software (version 6.0, https://mediacy.com/products/image-pro-plus/). Briefly, the loaded image was characterized using particle measurement section. LDs stained with Nile red were recognized by setting the threshold limits within 81 to 255. The selected region was further separated from neighbor by applying watershed module and was then automatically sized. The analysis was listed in “View measurement section”.

The colocalization of Plin4 and LD was automatically analyzed by Imaris software (version 9.0.1, https://imaris.oxinst.com/) using the *coloc* module. The colocalization was quantified by coloc-IntensitySum/Plin4-IntensitySum.

### LD extract

Adipocytes were isolated from inguinal fat pads by collagenase digestion as described (48). Briefly, WATs from mouse were cut into small pieces, and then incubated (1 g tissue/ml medium) in Krebs Ringer bicarbonate-Hepes buffer; 120 mM NaCl, 4 mM KH_2_PO_4_, 1 mM MgSO_4_, 1 mM CaCl_2_, 10 mM NaHCO_3_, and 27 mM Hepes, pH 7.4) containing 4% BSA and 0.5 mg/ml of collagenase type I (Worthington Biochemical) for 60 min at 37 °C. The suspension of cells was centrifuged for 2 min at 400*g*. The sediment was discarded. Dissociated adipocytes were filtered through nylon mesh (BD Falcon; BD) and then rinsed four times with Krebs Ringer bicarbonate-BSA buffer.

LDs were isolated from adipocytes as described (56). Briefly, adipocytes were resuspended in buffer A (20 mM tricine, 250 mM sucrose supplemented with 0.2 mM PMSF). After incubation on ice for 20 min, the cells were disrupted on ice ten times with a loose-fitting Dounce. After centrifuge at 3000*g* for 10 min at 4 °C, 10 ml of the postnuclear supernatant was collected and loaded with 2 ml of buffer B (20 mM Hepes, 100 mM KCl, 2 mM MgCl) on top. The samples were centrifuged at 2000*g* for 30 min at 4 °C. LDs from the top band were collected and were washed with buffer B for three times. Then the LD fractions were delipidated as described previously (57), and proteins were solubilized in SDS sample buffer for Western blot analysis.

### Statistical analysis

The GraphPad Prism software (version 8.0.0, https://www.graphpad.com/) was used to analyze and plot all data. The D’Agostino and Pearson omnibus normality test was used to determine the normal distribution of data. For data with a sample size smaller than 8, the Shapiro–Wilk test was considered to determine the distribution. For data with a normal distribution, single comparisons were performed by the two-tailed Student’s *t* test, whereas multiple comparisons were performed by one-way ANOVA with the Tukey post hoc test. For datasets that did not follow a normal distribution, single comparisons were performed by the Mann–Whitney test, and multiple comparisons were performed by the Kruskal–Wallis test with Dunn’s post hoc test. *p* values (<0.05) indicated a significant difference. Values of growth curve and colocalization analysis were expressed as the mean ± SEM. Values in other experiments were expressed as the mean ± SD.

## Data availability

The MS proteomics data have been deposited to the ProteomeXchange Consortium (http://proteomecentral.proteomexchange.org) *via* the PRIDE partner repository with the dataset identifier PXD043479. Other data in this study have been provided within the manuscript or the Supporting information.

## Supporting information

This article contains supporting information (58, 59).

## Conflict of interest

The authors declare that they have no conflicts of interest with the contents of this article.
