# Supplementary material for: Senp7 deficiency impairs lipid droplets maturation in white adipose tissues via Plin4 deSUMOylation
Source: J Biol Chem. 2024 Apr 25;300(6):107319. doi: 10.1016/j.jbc.2024.107319 (PMC11134554; doi:10.1016/j.jbc.2024.107319)
Supplement: Figures S1–S7 legends [file mmc1.docx]

**Supporting information figure legends**

**Supporting figure S1.**

1. Targeting strategy for the disruption of the *Senp7* gene.
2. The expression of *Senp7* in different tissues and organs from WT and *Senp7* KO mice was analyzed by real-time quantitative PCR. Expression levels of target genes were normalized to *Rplp0* (alias *36b4*) and data were normalized to WT group of each experiment. n = 4; mean ± SEM; *** *p* < 0.001 by two-tailed *t* test.

(C) Lysate of iWAT was immunoblotted (IB) with anti-Senp7 and anti-α-tubulin antibody. Star symbol indicated the band of Senp7.

(D) Body composition of male WT and *Senp7* KO mice measured by DEXA after 14 weeks on a regular chow diet. n = 5; mean ± SD; * *p* < 0.05, ** *p* < 0.01; ns, no significance by two-tailed *t* test.

(E) Total DNA content of whole iWAT and gWAT. n = 3; mean ± SD; ns, no significance by two-tailed *t* test.

(F) H&E staining of BAT sections from 14-week-old female WT and *Senp7* KO mice. Scale bars, 50 μm.

(G) Weight normalized to body weight of liver of 14-week-old female WT and *Senp7* KO mice. n = 3; mean ± SD; ns, no significance by two-tailed *t* test.

(H) H&E staining of liver sections from 14-week-old female WT and *Senp7* KO mice. Scale bars, 100 μm.

(I-J) Serum triglyceride level of 14-week-old male and female WT and *Senp7* KO mice. n = 8; mean ± SD; ns, no significance by two-tailed *t* test.

Error bars are represented as mean ± SD. * p < 0.05, ** p < 0.01, *** p < 0.001.

**Supporting figure S2.**

1. F) Indirect calorimetry of 14-week-old male WT and *Senp7* KO mice. (A) Feed: food intake. (B) Total activity. (C) Heat: heat generation. (D) VO_2_: oxygen consumption. (E) VCO_2_: CO_2_ generation. (F) RER: respiration exchange rate, VCO_2_/ VO_2_. WT n = 7, *Senp7* KO n = 5.

(G-I) Indirect calorimetry of 14-week-old female WT and *Senp7* KO mice. (G) VO_2_: oxygen consumption. (H) VCO_2_: CO_2_ generation. (I) RER: respiration exchange rate, VCO_2_/ VO_2_. n = 7. The column chart represents an average value during the light cycle (8:00~20:00) and dark cycle (20:00~8:00). Data are represented as mean ± SD; * *p* < 0.05; ns, no significance by two-tailed *t* test.

**Supporting figure S3.**

(A) The expression of *Senp7* in different tissues and organs from *Senp7*^fl/fl^ and *Senp7* AKO mice was analyzed by real-time quantitative PCR. Expression levels of target genes were normalized to *Rplp0* (alias *36b4*) and data were normalized to WT group of each experiment. n = 4; mean ± SEM; * *p* < 0.05; ns, no significance by two-tailed *t* test.

(B) Lysate of iWAT was immunoblotted (IB) with anti-Senp7 and anti-α-tubulin antibody. Star symbol indicated the band of Senp7.

(C) Body composition of male *Senp7*^fl/fl^ and *Senp7* AKO mice measured by DEXA after 14 weeks on a regular chow diet. n = 5; mean ± SD; * *p* < 0.05; ns, no significance by two-tailed *t* test.

(D) Total DNA content of whole iWAT and gWAT. n = 3; mean ± SD; ns, no significance by two-tailed *t* test.

(E) H&E staining of BAT sections from 14-week-old female *Senp7*^fl/fl^ and *Senp7* AKO mice. Scale bars, 50 μm.

(F) Weight normalized to body weight of liver of 14-week-old female *Senp7*^fl/fl^ and *Senp7* AKO mice. n = 3; mean ± SD; ns, no significance by two-tailed *t* test.

(G) H&E staining of liver sections from 14-week-old female *Senp7*^fl/fl^ and *Senp7* AKO mice. Scale bars, 100 μm.

(H-I) Serum triglyceride level of 14-week-old male and female *Senp7*^fl/fl^ and *Senp7* AKO mice. n = 8; mean ± SD; ns, no significance by two-tailed *t* test.

(J-L) Indirect calorimetry of 14-week-old female *Senp7*^fl/fl^ and *Senp7* AKO mice. (J) VO_2_: oxygen consumption. (K) VCO_2_: CO_2_ generation. (L) RER: respiration exchange rate, VCO_2_/ VO_2_. n = 8. The column chart represents an average value during the light cycle (8:00~20:00) and dark cycle (20:00~8:00). Error bars are represented as mean ± SD; * *p* < 0.05; ns, no significance by two-tailed *t* test.

**Supporting figure S4.**

(A-G) The level of alanine transaminase (ALT), aspartate transferase (AST), triglyceride (TG), cholesterol (CHOL), high-density lipoprotein cholesterol (HDL-CHOL), low-density lipoprotein cholesterol (LDL-CHOL), and non-esterified fatty acids (NEFA) in plasma of female mice fed with chow diet or HFD for 12 weeks checked by clinical blood chemistry analysis. Senp7^fl/fl^-Chow, n = 5; Senp7 AKO-Chow, n = 7; Senp7^fl/fl^-HFD, n = 8; Senp7 AKO-HFD, n = 8. Comparisons were performed between *Senp7*^fl/fl^ and *Senp7* AKO mice fed with each diets. Mean ± SD; * *p* < 0.05, ** *p* < 0.01 by two-tailed *t* test.

(H) Images of nile red-stained differentiated primary preadipocytes on day 30. Scale bars, 20 μm.

(I) Quantification of diameter of the largest LDs in each cell. n = 240 on day16; n = 360 on day 30; mean ± SD; *** *p* < 0.001 by two-tailed *t* test.

(J) Average volume of total LD in each primary preadipocyte which was differentiated for indicated days (n = 6 on day 16; n = 9 on day 30). Mean ± SD; * *p* < 0.05, ** *p* < 0.01 by two-tailed *t* test.

Data are represented as mean ± SD. * p < 0.05, ** p < 0.01, *** p < 0.001; ns, no significance.

**Supporting figure S5.**

1. SUMO2/3-conjugated proteins in WT and *Senp7* KO mice WAT were IP with anti-SUMO2+3 antibody, and the mmunoprecipitated proteins were blotted with anti-Plin1. Cell lysate was IB with anti-SUMO2+3 and anti-Plin1 antibody.

(B) SUMO2/3-conjugated proteins in WT and *Senp7* KO mice WAT were IP with anti-SUMO2+3 antibody, and the mmunoprecipitated proteins were blotted with anti-Plin2. Cell lysate was IB with anti-SUMO2+3 and anti-Plin2 antibody.

(C) SUMO2/3-conjugated proteins in WT and *Senp7* KO mice WAT were IP with anti-SUMO2+3 antibody, and the mmunoprecipitated proteins were blotted with anti-Plin3. Cell lysate was IB with anti-SUMO2+3 and anti-Plin3 antibody.

(D) SUMO2/3-conjugated proteins in WT and *Senp7* KO iWAT-1 cells were IP with anti-SUMO2+3 antibody, and SUMO-Plin4 proteins were blotted with anti-Plin4. Cell lysate was IB with anti-SUMO2+3 and anti-Plin4 antibody.

(E) Schematic diagrams of mice Plin4. Different Plin4 constructs include full-length Plin4 and Plin4-4mer.

(F) Helical wheel plot of one 33-mer repeat from Plin4, plottedas a 3–11 helix. Reported SUMOylated lysine residue is indicated (58).

(G) Weblogo generated from an alignment of 33-mer repeats from human Plin4 sequence (59).

(H) HeLa cells were transfected with 3xFlag-Senp7, 3xFlag-Senp7-C979S, or HA-SUMO2+3 as indicated. Cell lysate was immunoblotted with anti-HA antibody (top panel) and anti-α-tubulin antibody (bottom panel).

**Supporting figure S6.**

(A) The expression of *Plin4* in gonadal fat from WT and *Senp7* KO mice was analyzed by real-time quantitative PCR. Expression levels of target genes were normalized to *Rplp0* (alias *36b4*) and data were normalized to WT value. n = 3; mean ± SEM; * *p* < 0.05, ** *p* < 0.01, *** *p* < 0.001; ns, no significance by two-tailed *t* test.

(B-C) The expression of Plin4 in gonadal fat from WT and *Senp7* KO mice was analyzed by western blot. Tissue lysates were immunoblotted with anti-Plin4 antibody (top panel) and anti-α-tubulin antibody (bottom panel). (C) The gray scale value was normalized to WT value. The value of gray scale was analyzed by Image J. n = 6; mean ± SD; * *p* < 0.05, ** *p* < 0.01, *** *p* < 0.001; ns, no significance by two-tailed *t* test.

(D) The expression of *Plin4* during primary preadipocyte differentiation was analyzed by real-time quantitative PCR. Expression levels of target genes were normalized to *Rplp0* (alias *36b4*) and data were normalized to WT value on day 0 of each experiment.

(E) The expression of Plin4 during primary preadipocyte differentiation was analyzed by western blot. Cell lysates were immunoblotted with anti-Pin4 antibody (top panel) and anti-α-tubulin antibody (bottom panel).

(F-G) *Senp7* and *Plin4* in iWAT-1 cells were knocked down using siRNA. The expressions of *Senp7* and *Plin4* were analyzed by real-time quantitative PCR. Expression levels of target genes were normalized to *Rplp0* (alias *36b4*) and data were normalized to WT value of each experiment. n = 3; mean ± SEM; * *p* < 0.05, ** *p* < 0.01 by two-tailed *t* test.

(H-J) LD fractions were isolated from iWAT derived from WT and Senp7 KO mice. LD proteins were immunoblotted with anti-Plin4, or anti-Plin1 antibody.

(K) *Senp7* in iWAT-1 cells were knockout using CRISPR/Cas9 strategy. Monoclonal cell lines were generated and were named by the clonal number. The expressions of *Senp7* in indicated cell lines were analyzed by real-time quantitative PCR. Expression levels of target genes were normalized to *Rplp0* (alias *36b4*) and data were normalized to negative control (N. C.) value of each experiment. Values of KO strains were compaired to values of N. C. cells. n = 3; mean ± SEM; * *p* < 0.05, ** *p* < 0.01, *** *p* < 0.001; ns, no significance by two-tailed *t* test.

(L) Senp7 KO #44 iWAT-1 cells were transfected with Plin4 by electroporation as indicated. Cell lysate was immunoblotted with anti-SUMO2+3 antibody (top panel) and anti-α-tubulin antibody (bottom panel). SUMO2/3-conjugated proteins in iWAT-1 cells were IP with anti-SUMO2+3 antibody, and SUMO-Plin4 proteins were blotted with anti-Plin4. Cell lysate was IB with anti-Plin4 antibody (second panel), anti-Senp7 antibody (third panel), anti-SUMO2+3 antibody (forth panel), and anti-α-tubulin antibody (bottom panel).

(M) WT and *Senp7* KO iWAT-1 cells were transfected with 3xFlag-Senp7, or 3xFlag-Senp7-C979S as indicated. Cell lysate was immunoblotted with anti-Flag antibody (top panel) and anti-α-tubulin antibody (bottom panel).

Data are represented as mean ± SD. * p < 0.05, ** p < 0.01.

**Supporting figure S7.**

1. Lipid exchange efficiency was analyzed by FRAP. LD images in live differentiated primary preadipocytes on day 8 were captured manually. The yellow circle represents the photobleached area. Scale bars, 5 μm.
2. MOI percentage in the bleached (yellow circle) or unbleached (blue circle) region was obtained from six independent experiments.
