# Supplementary material for: Senp7 deficiency impairs lipid droplets maturation in white adipose tissues via Plin4 deSUMOylation
Source: J Biol Chem. 2024 Apr 25;300(6):107319. doi: 10.1016/j.jbc.2024.107319 (PMC11134554; doi:10.1016/j.jbc.2024.107319)

Supporting figure S1.


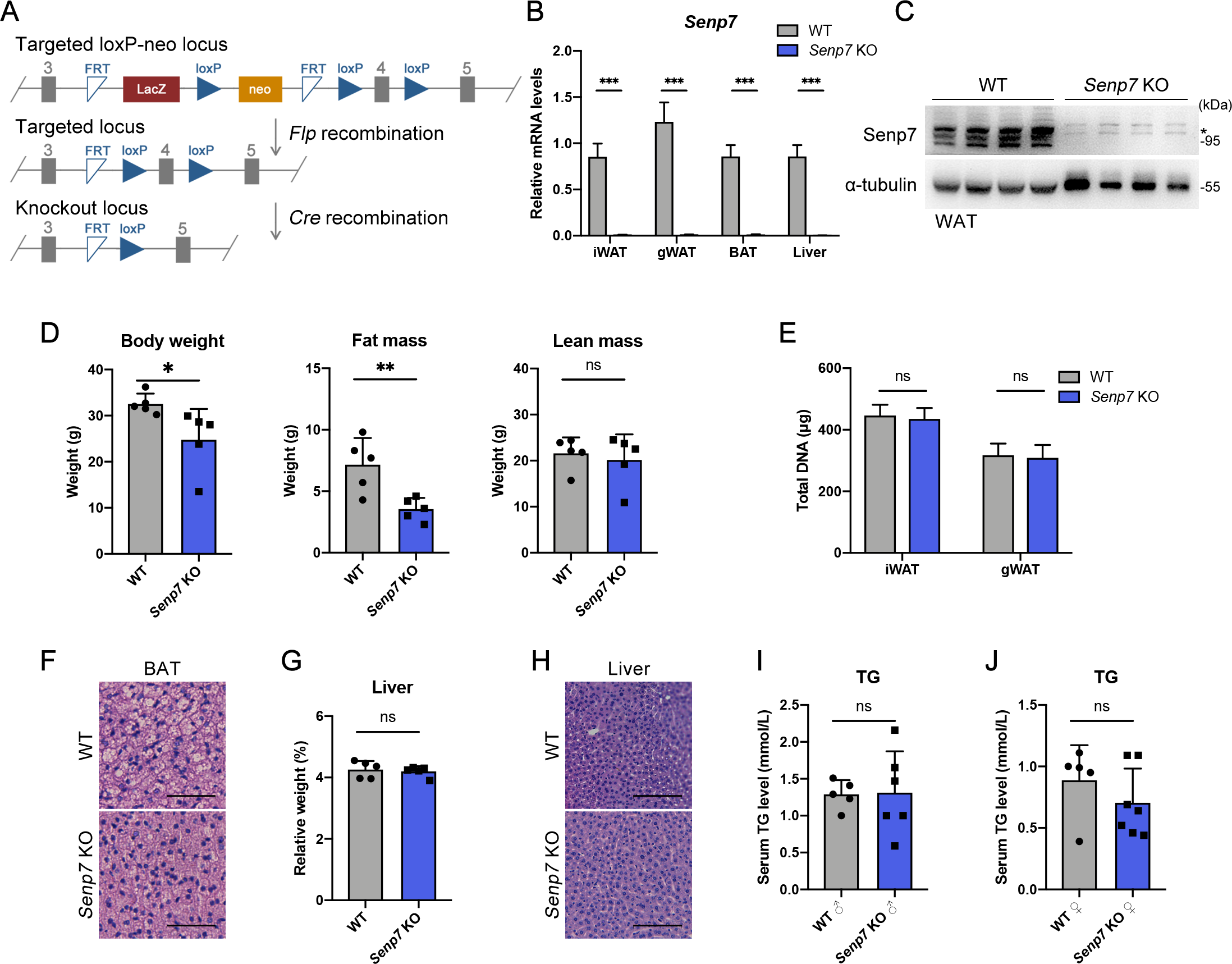


Supporting figure S2.


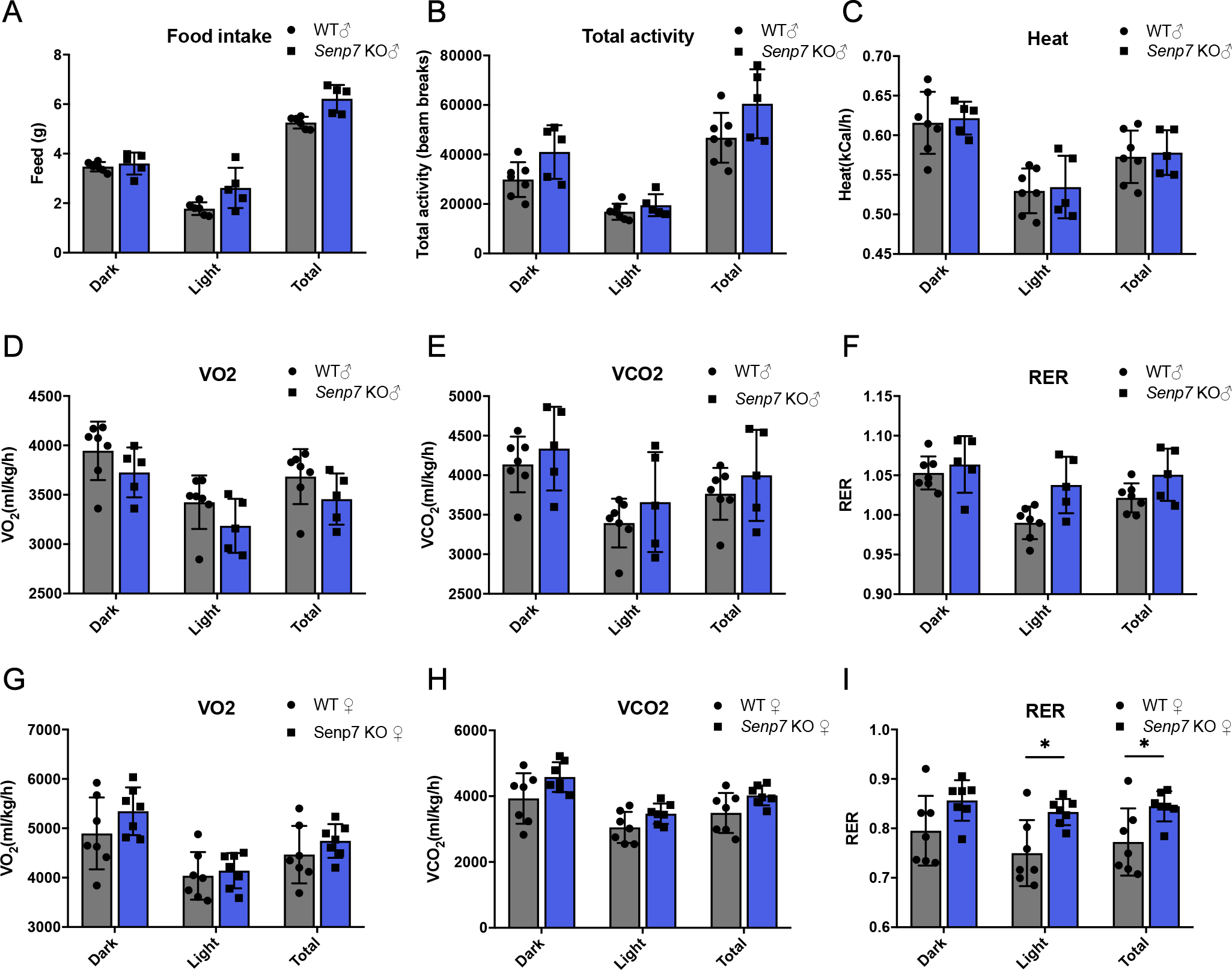


Supporting figure S3.


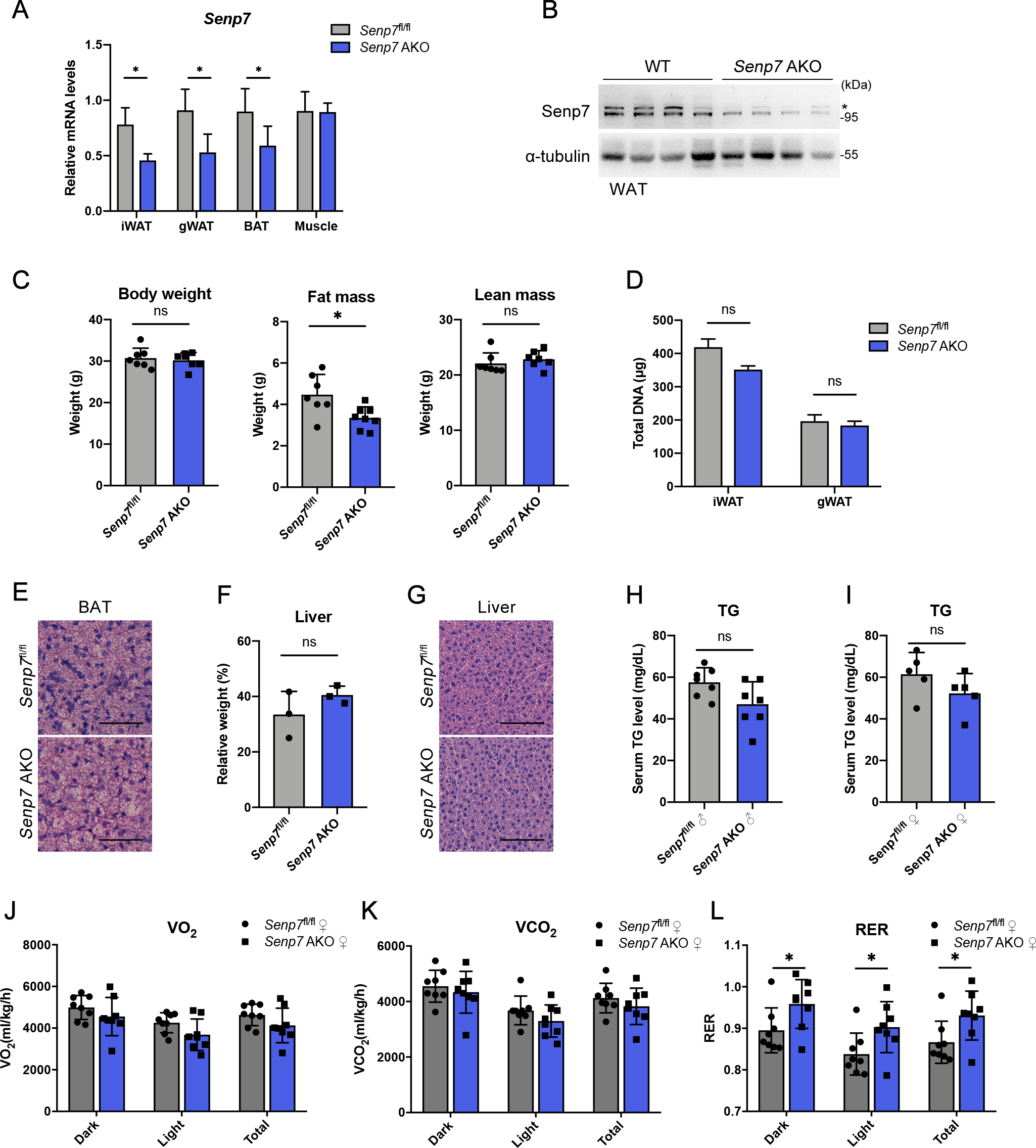


Supporting figure S4.


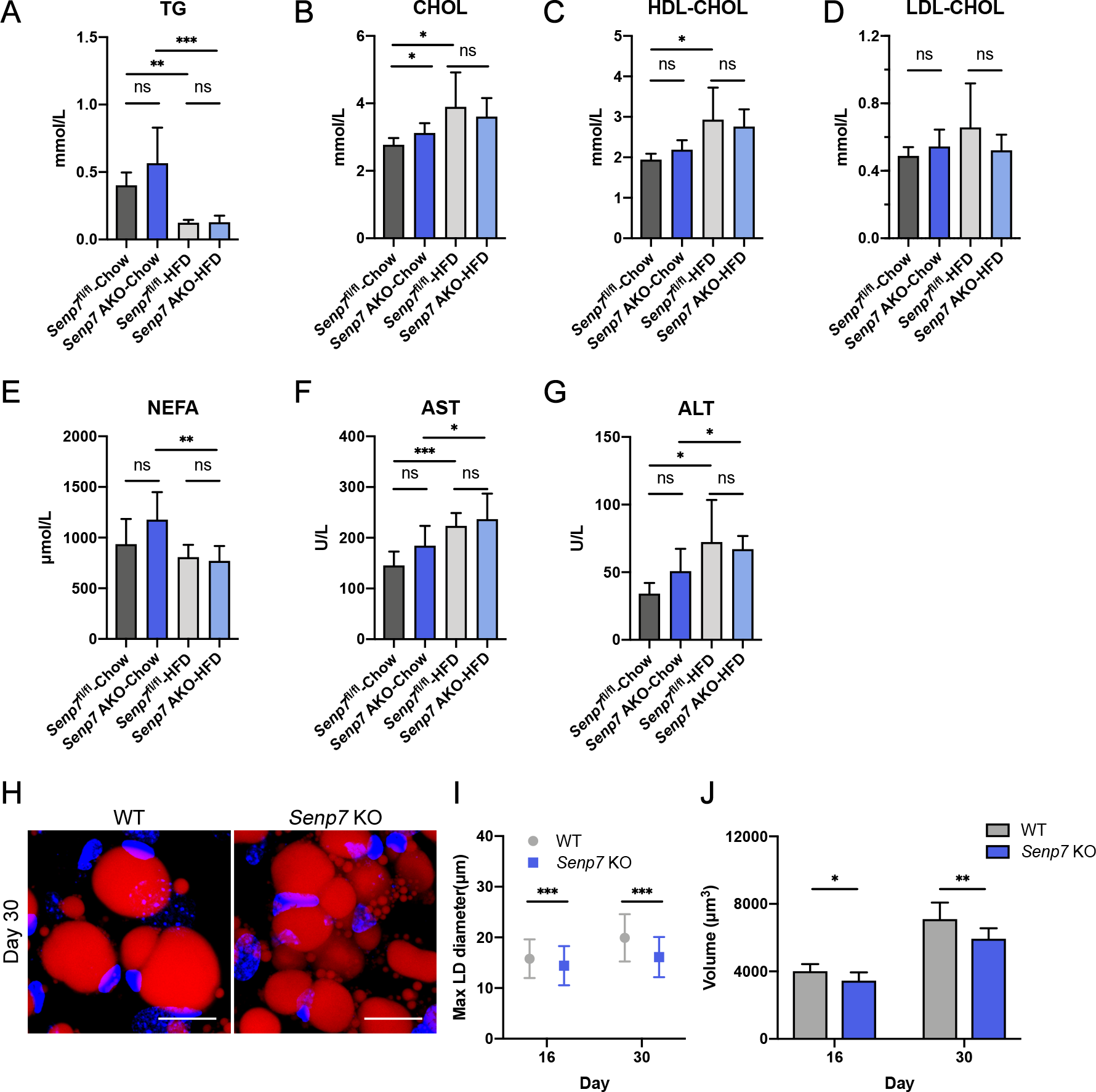


Supporting figure S5.


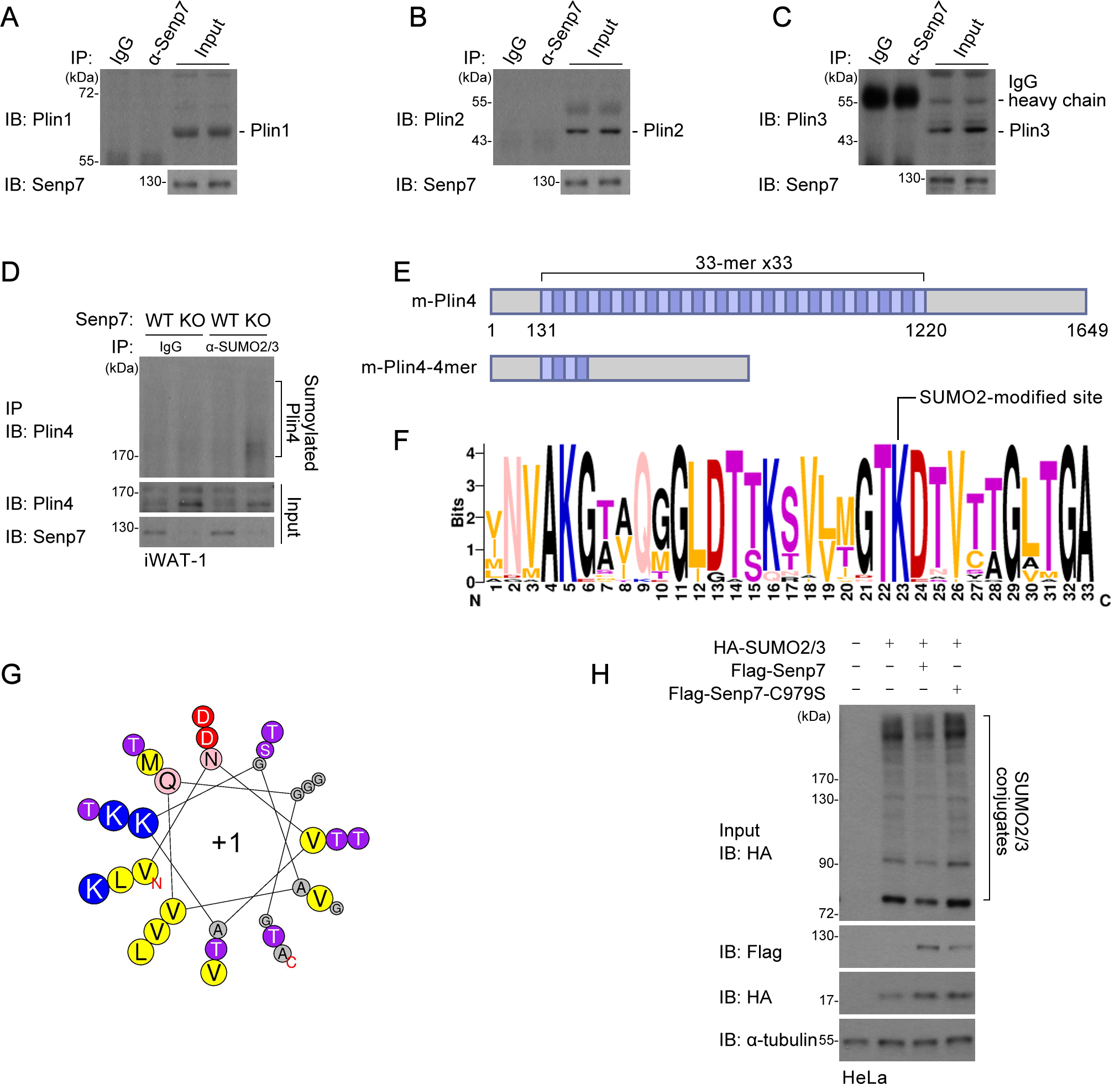


Supporting figure S6.


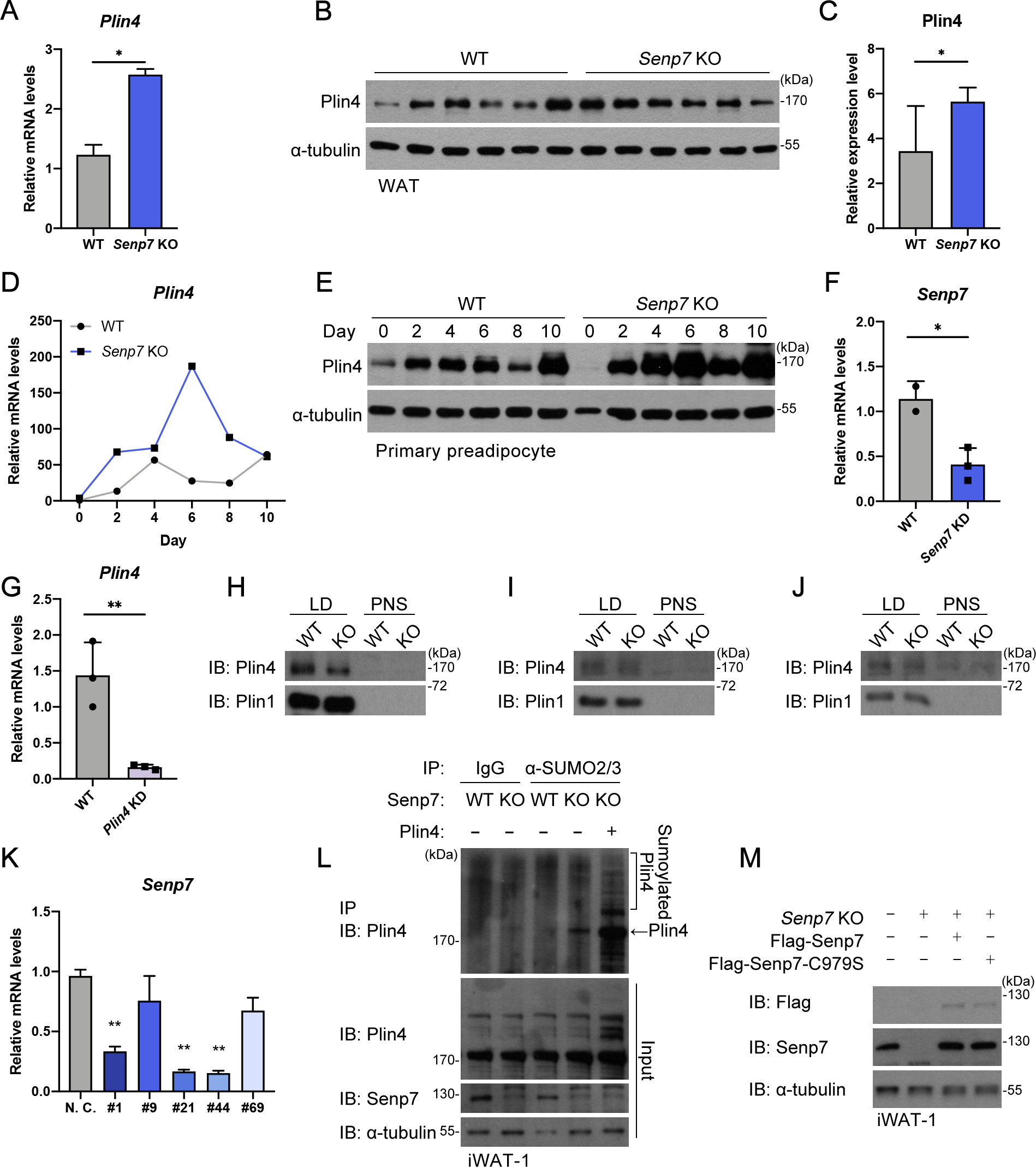


Supporting figure S7.


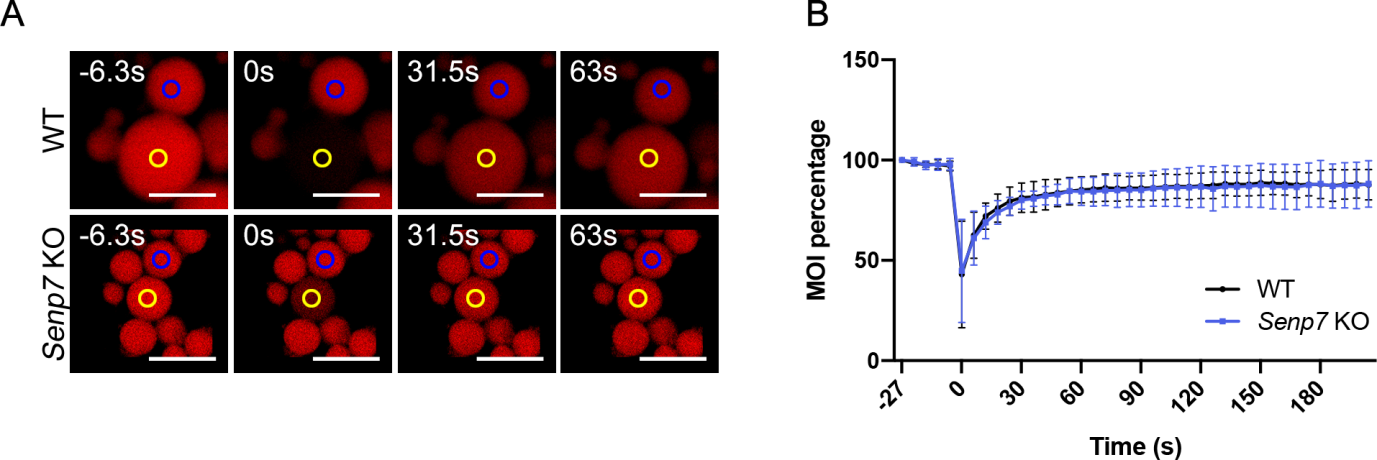

Supplement: Figures S1–S7 [file mmc2.docx]
